# Biodiversity impacts due to food consumption in Europe

**DOI:** 10.1016/j.jclepro.2019.04.054

**Published:** 2019-08-01

**Authors:** E. Crenna, T. Sinkko, S. Sala

**Affiliations:** European Commission, Joint Research Centre (JRC), Via Enrico Fermi 2749, I-21027, Ispra, Italy

**Keywords:** Biodiversity, LCA, Impact assessment, Food production and consumption, Integrated assessment

## Abstract

Food security and biodiversity conservation are closely interconnected challenges to be addressed to achieve a sustainable food system on a global scale. Due to the complex nature of food production and consumption system, quantifying the impacts of food supply chains on biodiversity is challenging. Life cycle assessment (LCA) allows for systematically addressing environmental impacts along supply chains, representing a reference methodology that can be applied for assessing food systems. In the present study, 32 representative food products of consumption in the European Union (EU) were selected and their environmental impacts calculated through a process-based LCA. The potential contribution of EU food consumption to the current biodiversity decline has been evaluated adopting both midpoint and endpoint indicators. A comparison of the impact drivers was performed. Meat products, the underpinning land use for agricultural purposes, and climate change represent the main hotspots of impacts on biodiversity. Notwithstanding several drivers of biodiversity loss can be accounted for with LCA, the evidence of the increasing biodiversity decline on both a European and a global scale indicates that the assessment system should be further expanded, especially for what concerns refining impact categories such as ecotoxicity, and including resource overexploitation, and impact due to invasive species. This study illustrates: how far the current LCA based impact assessment framework may help to address the drivers of biodiversity loss; which are the main uncertainties associated to results stemming from the application of different endpoint methods; which aspects need to be elaborated further to ensure a comprehensive assessment of biodiversity impacts due to food production and consumption.

## Highlights

• Assessment of representative food products consumed in EU and driving biodiversity loss.
• Environmental impacts of EU food consumption through a product-based LCA.
• Assessment of biodiversity impacts of EU food consumption (endpoint analysis).
• Meat products and agricultural land use as main hotspots of impact for biodiversity.
• Key limitations for biodiversity impact assessment in LCA.

## Graphical abstract

**Figure:**
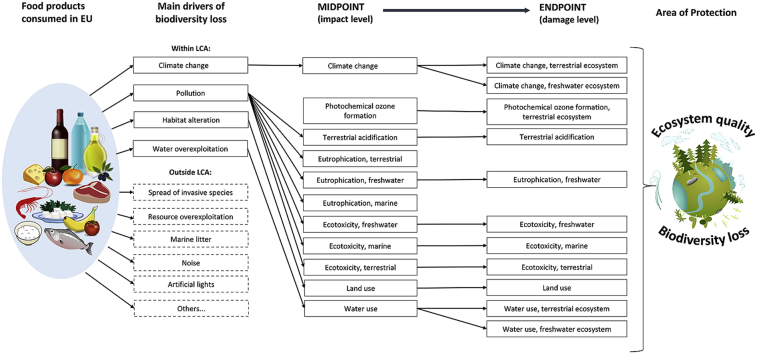

## 1 Introduction

Food security represents one of the main sustainability challenge, listed among the Sustainable Development Goals (SDG 2) (UN, 2015). Meeting the future needs of an increasing human population while remaining within the limits of our planet (EU, 2013), requires the evolution of food systems, with a transition towards environmental sustainability. In fact, food production and consumption is amongst the major drivers of environmental degradation (Notarnicola et al., 2017) and biodiversity decline (Chaudhary and Kastner, 2016). This calls for a responsible and sustainable production and consumption (SDG 12) all along the different steps of the food supply chains. For example, sustainable agriculture should be able to respond to human needs while decreasing the associated environmental pressures and impacts, ultimately those on biodiversity. Indeed, food security and biodiversity conservation are closely interconnected challenges (Frison et al., 2011; Fischer et al., 2017). Food production is generating great pressure on the environment, while being substantially dependent on biodiversity and on the services provided by ecosystems. There is an increasing evidence that as long as biodiversity sustaining food production and agriculture declines, the supply of food becomes more vulnerable and unsustainable (Sundar, 2011; Crist et al., 2017).

Achieving the SDGs and the Aichi Biodiversity Targets (CBD, 2016) requires a systematic assessment and improvement of supply chains. Life Cycle Assessment (LCA) is a pivotal methodology to analyse supply chains and to identify hotspots and trade-offs of environmental impact. Several LCA studies have been focusing on the assessment of the food system, in many cases stating that there are still limitations to be overcome (Sala et al., 2017).

The majority of the studies related to food consumption in LCA has been conducted adopting midpoint indicators, focusing on climate change, acidification and eutrophication (e.g. McClelland et al., 2018). Recent studies link midpoint and endpoint, focusing on biodiversity loss due to e.g. land use (Chaudhary and Kastner, 2016) and water use (Scherer and Pfister, 2016). Studies to assess the role of food consumption on biodiversity decline on a global scale were conducted with either a bottom-up or a top-down approach, namely adopting either process-based LCA or multiregional input-output analysis (MRIO) combined with LCA.

For instance, Wolff et al. (2017) and Chaudhary and Kastner (2016) carried on a LCA study observing that agricultural products contribute to different extents to biodiversity decline. Chaudhary and Kastner (2016) focused on the impacts of agricultural products and identified wheat, rice, and maize (due to high land occupation), as well as sugarcane, palm oil, coconut, cassava, rubber, and coffee (due to high species richness in the places where they are cultivated, although low land occupation at global level) among the major impacting products on biodiversity. In line with the previous studies, through a MRIO model, Lenzen et al., (2012) identified coffee, cocoa, palm oil and coconut - which e.g. in Papua New Guinea are linked to nine critically endangered species- as contributors to biodiversity decline especially in developing countries. By coupling traditional LCA with MRIO, Scherer and Pfister (2016) quantified biodiversity impacts in Switzerland and in other commercially connected countries. The authors mainly observed that biodiversity of several countries is affected by Swiss food consumption, including Ecuador, USA and Spain from where coffee, cocoa, and almonds are imported.

Beyond the LCA application, there are comprehensive studies conducted in the biodiversity conservation domain to highlight the role of food in the biodiversity decline. For example, Donald (2004) summarized the existing literature concerning the impacts of low- and high-intensity food production systems (e.g. cocoa, coffee, rice, palm oil, and soybean) on the natural environment, highlighting the potential loss of habitats and the decline in several taxa (e.g. forest-specialist birds). The same concerns are highlighted in more recent studies, e.g. Middendorp et al. (2018) on cocoa and Kwatrina et al. (2018) on palm oil.

When assessing the impacts on biodiversity, the focus is generally on the transformation of natural land and its occupation for agricultural purposes or urbanization, as habitat change represents one of the main sources of biodiversity loss (MEA, 2005; Barnosky et al., 2011). Nevertheless, it is acknowledged that also other impacts related to food production and consumption systems have to be taken into account as they drive biodiversity decline. For instance, fish aquaculture not only needs a substantial amount of feed, thus causing high pressures on terrestrial ecosystems, but also generates nutrient emissions due to manure and feed spill, potentially leading to water eutrophication (Ellingsen et al., 2009; Junior et al., 2018).

In literature, bottom-up studies often assess one food product or category at the time. Few recent studies, as the Basket of Products (BoP) indicators (EC-JRC, 2012) and Notarnicola et al. (2017), have widely addressed the environmental pressures of food consumption covering a number of representative products and their related supply chains by adopting a life cycle approach.

The novelty of the present study lies in performing a bottom-up LCA focusing on the food consumption in the European Union (EU) covering a wide range of food products, and addressing biodiversity impacts. The impacts are assessed by means of different models which are compared and critically discussed, showing limitation and knowledge gaps in light of the available knowledge in literature.

More in details, the present study aims to:

(i) improve the work previously done by Notarnicola et al. (2017), expanding the environmental assessment from 19 to 32 representative food products;
(ii) evaluate the role of EU food consumption in the current biodiversity decline, presenting results of midpoint and endpoint modelling;
(iii) unveil biodiversity loss drivers currently not captured by LCA modelling, which should be addressed in future research.

Section 2 presents the method adopted for identifying the additional representative food products and assessing their environmental impacts both at midpoint and endpoint, with a focus on biodiversity. Results are presented in section 3, followed by a discussion on the limitations and gaps in LCA regarding biodiversity assessment in section 4. Finally, conclusions are drawn in section 5.

## 2 Material and methods

The assessment of impacts of EU food production and consumption on biodiversity has been performed as follows:

(i) selection and modelling of representative food products, complementing the list of products in Notarnicola et al. (2017) with those recognized for their alleged impacts on the environment (Section 2.1);
(ii) characterization of impacts both at midpoint and endpoint level (Section 2.2)

### 2.1 Selection and modelling of the representative food products

This paper is based on the study of the Basket of Food Products (BoP Food) developed in Notarnicola et al. (2017), later expanded by Castellani et al. (2017). The studies included 19 representative food products that were selected for their importance in the food system in terms of either mass or economic value. However, also products with small consumption amount or low economic value can have significant impacts e.g. on biodiversity loss, as shown in the literature (Table 1). Drivers of impacts are mainly related to water use, land occupation and transformation, e.g. for monoculture cultivation. All these drivers may lead to loss of habitat and species richness, from mammals to insects and plants. In addition, pesticide use is a hotspot related to many food products, leading also to degradation of habitat, and decline of several taxa, including e.g. amphibians and pollinators populations. Specific impacts are related to resource overexploitation, e.g. overfishing, which is directly affecting the availability and renewability of fish stocks.

**Table 1:** Examples of food products consumed in EU and theirassociated pressure and impacts, especially on biodiversity.

Product	Pressures generated by human interventions	Impacts on natural environment and target taxa
Beef/pork/poultry meat	Land use change, e.g. in Brazil^a^ or in Europe, from natural areas into monoculture of soybean (also used for feeding bred animals)	Loss of habitats suitable for endangered species, e.g. black-faced lion tamarin and ring-tail monkey^b^, or for helpful insects, birds and bats as pollinators
Dairy products		
Tofu		
Salmon		
Eggs		
Oils		
Biscuits (palm oil)	Land use change in Indonesia^c^, from natural areas into palm oil monoculture plantations	Loss of habitats suitable for endangered species, e.g. gibbons and Javan rhinoceros^d^
Tea	Pesticide use^e^	Contamination of water courses and habitat degradation, leading to amphibian, bird and pollinator populations' decline^f^
Chocolate (cocoa beans)		
Tomatoes		
Fruit cultivation, e.g. apples, oranges, grapes for wine		
Banana		
	Land use change, e.g. in Latin America, from natural areas into monoculture banana plantations g	Loss of habitats suitable for several species, from insects to mammals k
Rice	High land occupation, e.g. in Europe and Mediterranean areas^h^	
	Water use^i^	
Almond		
Cod	Sea bottom trawling j	
	Overfishing^l^	Loss of wild cod stock^l^ and disruption of the trophic chain
Shrimps	Nutrient emissions^m^	Excessive algae blooms^n^, with eutrophication of marine and fresh water, thus leading to species composition change and disruption of the trophic chain
	Conversion of tropical coastal lowlands of America and Asia into shrimp ponds o	Habitat degradation and loss, especially salt marshes and mangrove areas o
Agricultural commodities	Spread of invasive species due to commodities trade, e.g. pathogens from China and the USA p	Loss of helpful insects (e.g. pollinators), thus affecting food security

a E g. Caro et al. (2018)..

b EEC, 2018.

c Wicke et al. (2011).

d Rainforest Action Network (2018).

e Gurusubramanian et al. (2008) (tea), Ntiamoah and Afrane, 2008 (cocoa), Torrellas et al. (2012) (tomato), BananaLink (2018) (banana).

f Aktar et al. (2009).

g BananaLink (2018).

h Chaundhary and Kastner (2016).

i Blengini and Busto (2009) (rice), Scherer and Pfister (2016) (almond).

j Ellingsen et al. (2009).

k Winter et al. (2017).

l WWF, 2018.

m Cao et al. (2011).

n EPA, 2017.

o Páez-Osuna (2001).

p Paini et al. (2016).

Some products with known biodiversity impacts, as coffee, meat, and milk, have been already addressed by Notarnicola et al. (2017). Some other products like cocoa and palm oil (largely used in the confectionery industry), almonds, tea, and seafood were not yet included in the basket of representative food products consumed by EU citizens. These products mainly come from trade, imported from developing countries. The studies in literature converge on that developed countries are major net importers of embodied biodiversity loss, associated with commodities coming from developing countries.

Therefore, the work done by Notarnicola et al. (2017) and Castellani et al. (2017) was expanded by including 13 additional food products that complement the previous selection. The additional representative products were selected within the available literature based on the following criteria:

− their relevance in terms of environmental impacts, specifically on biodiversity: almonds, tea, shrimps, rice, chocolate (cocoa), biscuits (palm oil);
− highly imported products: bananas, cod, salmon;
− products that show new trends in nutrition (i.e. products used in a vegetarian diets as a protein source): tofu, beans;
− other relevant products to supplement the selection of food due to the consumed quantities: eggs, tomatoes.

The functional unit was defined as the average food consumption in the European Union and per EU citizen in terms of selected food categories in 2015. Table 2 reports the products included in the study and their apparent consumption in 2015 (= production within the EU boundaries + import - export). The annual consumption of representative products is 684 kg per capita per year, which represents 42% of the total annual apparent consumption.

**Table 2:** Composition of the food consumption assessed in this study in terms of product groups, representative products and related quantities referred to the reference flow, i.e. food consumption of an average EU citizen in the reference year 2015 (Eurostat, 2018). The 13 new products added in this study are shown in bold.

Product Group	Representative product	Per capita consumption in 2015 (kg/person. yr^−1^)	Share within included food
MEAT	Pork meat	44.9	6.6%
	Beef meat	15.2	2.2%
	Poultry meat	26.3	3.8%
**FISH & SEAFOOD**	**Cod**	**10.4**	**1.5%**
	**Salmon**	**3.5**	**0.5%**
	**Shrimps**	**1.5**	**0.2%**
DAIRY	Milk	78.4	11.5%
	Cheese	15.1	2.2%
	Butter	4.4	0.6%
**EGGS**	**Eggs**	**14.0**	**2.0%**
CEREAL-BASED PRODUCTS	Bread	40.0	5.8%
	Pasta	9.3	1.4%
	**Rice**	**9.6**	**1.4%**
SUGAR	Sugar	28.6	4.2%
OILS	Sunflower oil	5.7	0.8%
	Olive oil	4.7	0.7%
TUBERS	Potatoes a	68.5	10.0%
**VEGETABLES**	**Tomatoes**	**14.5**	**2.1%**
**LEGUMES**	**Beans**	**2.8**	**0.4%**
	**Tofu**^b^	**5.1**	**0.7%**
FRUITS	Apples	17.5	2.6%
	Oranges	13.0	1.9%
	**Bananas**	**11.5**	**1.7%**
**NUTS & SEEDS**	**Almonds**^a^	**0.6**	**0.1%**
COFFEE & TEA	Coffee	3.3	0.5%
	**Tea**	**0.6**	**0.1%**
BEVERAGES	Beer	70.0 L	10.2%
	Wine	26.0 L	3.8%
	Mineral water	122.3 L	17.9%
**CONFECTIONERY PRODUCTS**	**Biscuits**	**7.1**	**1.0%**
	**Chocolate**	**6.0**	**0.9%**
PRE-PREPARED MEALS	Meat based dishes	3.4	0.5%

a Based on 2013 data, 2015 data was not available.

b EEA (2018) data.

**Fig. 1:**
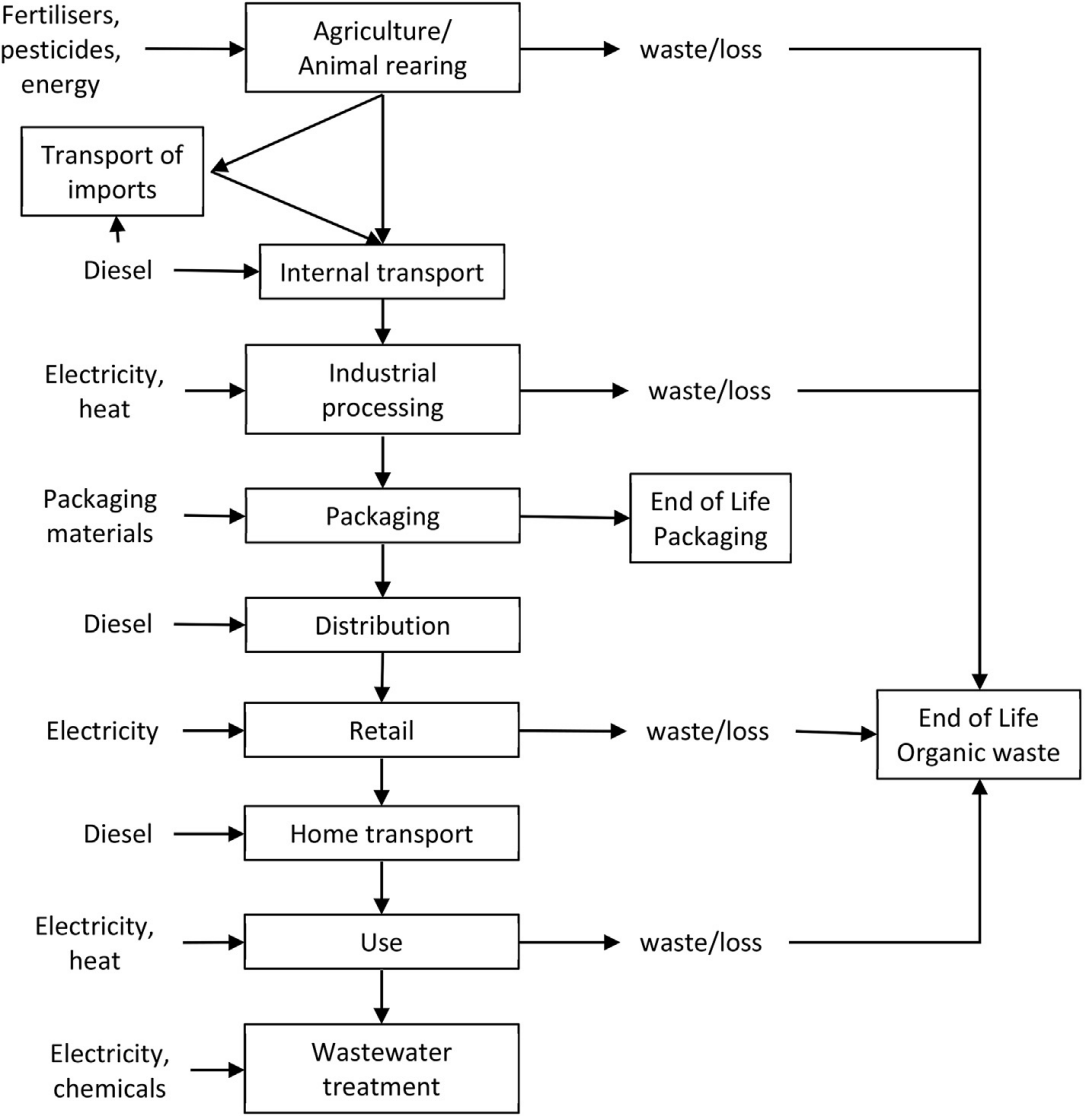
System boundary adopted for assessing the food products in this study.

The included life cycle stages related to all food products were agriculture, animal rearing, industrial processing, packaging, logistic, retail, use and end of life (Fig. 1). The main Life Cycle Inventory (LCI) data sources, such as e.g. Agrifootprint (Blonk Consultants, 2014) are complemented with specific data from literature. Indeed, for the different food products as far as possible LCI data related to cultivation or processing (e.g. fuels, electricity, pesticide), were retrieved from different LCA studies. For instance, input data for rice cultivation and processing are based on Blengini and Busto (2009). Salmon aquaculture data were derived from Pelletier et al. (2009), while input data for processing were taken from Elingsen et al. (2009). The detailed LCI data and related sources are presented in the supplementary material (SM, Tables S1-S9). Economic allocation was used, when needed. However, in case of salmon, no burdens were allocated to the co-products. The most representative datasets for each product in the BoP Food were identified from the existing LCA literature. All the agricultural and processing datasets were adapted to the average EU situation, when cultivation and processing were located in Europe.

Agricultural datasets were adapted with the same assumptions as made in Notarnicola et al. (2017), meaning that N_2_O emissions from managed soils, CO_2_ emissions from lime and urea application, NH_3_ emissions to air and nitrate leaching in soil were estimated according to the IPCC guidelines (IPCC, 2006). Namely, it was assumed that all nitrogen that volatizes converts to ammonia, while all nitrogen that leaches is emitted as nitrate. It was estimated that 5% of phosphorus applied through fertilisers is emitted to freshwater (Blonk Consultants, 2014). Pesticides (as active ingredients) were assumed to be emitted into soil (de Beaufort-Langeveld et al., 2003). Water use takes into account only blue water, i.e. water sourced from surface or groundwater resources, such as irrigation water in case of agriculture (Chapagain and Hoekstra, 2010).

Logistics consist of international transportation from outside the EU territory, transport of raw materials to the processing site, and transport of the processed goods from industry to retailing. For each kg of imported goods, the inventory of transport with ferry and truck was calculated considering the different exporting countries and distances. The shares of imported products and transportation distances are available in the supplementary material. The use of refrigerants, both their load and leakage, was included in the inventory of refrigerated/frozen transportation and storage related to the products that need to be refrigerated. The refrigerant R404A was considered, as it is the most commonly used refrigerant in Europe. The LCA data for the production of the refrigerants is from Bovea et al. (2007).

The use phase consists of the transportation of food from retail to consumer and domestic consumption. The assumption behind transportation is that all 32 food products are bought in a single purchase. In this way, the impact of transport is allocated between the products considering that each of them is one of thirty-two items purchased (3.13% of the transport burden for each food product). This choice, which is based on a simplification, namely assuming to give equal burden to each product, has been taken to be consistent with Notarnicola et al. (2017), according to the approach of Vanderheyden and Aerts (2014). The energy consumption in households is reported in the supplementary material.

The end of life (EoL) stage was modelled taking into account both burdens of waste management and potential benefits of recycling and reuse. The end-of-life phase includes packaging, treatment of food scraps and unconsumed foods (both avoidable and unavoidable), together with the environmental assessment of human metabolism products, modelled according to the method developed by Muñoz et al. (2007).

### 2.2 Characterization of the impacts due to EU food consumption at midpoint and endpoint

The life cycle assessment of the BoP Food was performed by using SimaPro software v.8.5 (Pré-Sustainability, 2018). The inventory was characterized both at midpoint and endpoint level, excluding all long-term emissions. At midpoint level, the Environmental Footprint (EF) (EF reference package 2.0) midpoint method (EC, 2017a) in the version EF reference package 2.0 (EC-JRC, 2018; Fazio et al., 2018) was used, including the 16 impact categories. At endpoint level, ReCiPe 2008 (Goedkoop et al., 2009) and its most up-to-date version ReCiPe 2016 (Huijbregts et al., 2016) were used. Specifically, the hierarchist perspective, which is based on scientific consensus with respect to the time horizon and the probability of impact mechanisms, was selected for both methods. The choice of using and comparing both methods is linked to: i) the different modelling approaches behind several indicators (e.g. land use, terrestrial acidification, etc.) reflecting the evolution of the scientific literature; ii) an update in number of impact categories, namely photochemical ozone formation and water consumption have been introduced in the more recent 2016 version of ReCiPe method.

Biodiversity loss is assessed by endpoint indicators, linking the different environmental impacts quantified by the midpoint impact categories to the Area of Protection (AoP) “ecosystem quality”, which represents one of the three issues of concern for human society. The endpoint indicators reflect the midpoint impact categories at a farther level of the cause-effect chain. As the midpoint categories, they are associated with different stressors, impact pathways and representative species (e.g. vascular plants for acidification; vascular plants, algae, freshwater fish species and invertebrates for eutrophication; vascular plants, mammals, birds and some arthropods such as butterflies for land use; freshwater fish species for water use, etc.) (Goedkoop et al., 2009; Huijbregts et al., 2016; Woods et al., 2017). Therefore, the figure for biodiversity loss derives from the sum of the results of the endpoint categories converging in the AoP “ecosystem quality”. The unit for ecosystem quality is Potentially Disappeared Fraction (PDF) of species over time, expressed as PDF*years. This metric measures the rate of species lost in a particular area of land or volume of water during a particular time following land transformation and occupation, emission of toxic substances, climate change, etc.

Since land use is recognized as one of the most relevant drivers of biodiversity loss (MEA, 2005; Barnosky et al., 2011), a specific focus on its damage to biodiversity was considered. The endpoint method developed by Chaudhary et al. (2015) was implemented in SimaPro software v.8.5 (Pré-Sustainability, 2018). The sets of so-called “world average” characterization factors (CFs), which consider the whole world as a unique region by assigning the magnitude of the impact without accounting for the spatial explicit location where the impact occurs, were applied to the BoP Food. In fact, due to a limitation in the spatial details of the BoP inventory, it was not possible to use the CFs spatially referred at country level proposed by Chaudhary et al. (2015). The method provides four sets of “world average” CFs, which reflect global (irreversible) extinctions or regional (semi-reversible) disappearances of species, and are based on either marginal or average impact assessment approach. To estimate the global extinctions, Species-Area Relationship (SAR) models –which predict species loss following habitat loss in a region– are combined with vulnerability scores derived from the International Union for Conservation of Nature (IUCN) criteria. The “world average” CFs in the method are provided per aggregated taxa, based on five taxonomic classes (i.e. mammals, birds, amphibians, reptiles and vascular plants).

All the land use models adopted in this study (ReCiPe 2008 based on De Schryver and Goedkoop (2009), ReCiPe 2016 based on both De Baan et al. (2013) and Curran et al. (2014), and Chaudhary et al. (2015)) are built on SAR models, although of different types: classical, matrix and countryside. The basic assumption behind the classical SAR model is that the areas converted by humans are completely hostile to biodiversity, potentially leading to overestimating species loss. On the other hand, the matrix SAR and the countryside SAR models, respectively adopted in ReCiPe 2016 and in the approach by Chaudhary et al. (2015), account for habitat heterogeneity, assessing patterns of species richness in multi-habitat. Unlike the classic or matrix SAR, the countryside SAR model recognizes the fact that species can adapt to human-dominated habitats and still survive in the absence of natural habitat.

Given the complexity of biodiversity impact assessment and the rather limited possibility of apprasing them completely, it is important to stress what the above-mentioned biodiversity impact indicators aim at measuring.

Firstly, to understand the results and interpret them correctly, it has to be acknowledge that these models are assessing potential impacts to biodiversity, by means of modelling to which extent environmental pressures are potentially leading to biodiversity loss. Moreover, biodiversity impacts vary largely depending on location and actual affected ecosystems. In these approaches, the level of details in terms of spatial explicit location where the impact occurs is very coarse. As the location greatly influences biodiversity loss, the potential impacts calculated should be interpreted with caution.

The approaches used here are considered the best possible, considering that spatially explicit data on where the impact occur is not available in detail. However, they rather measure a risk/pressure intensity than a factual biodiversity loss (e.g. a ton of reactive N lost to waterbodies has hugely diverse impacts depending on where it occurs). The approaches based on matrix and landscape SAR are attempting to account for this, by modelling the link between specific habitat and composition of species and to improve the spatial differentiation of impacts.

## 3 Results

The results of the assessment are illustrated henceforth: firstly, the environmental impacts of EU food consumption at midpoint, characterised with the EF 2.0 method; then, the impacts of EU consumption on biodiversity, with a focus on the impacts due to land use.

### 3.1 Overall environmental impact of EU food consumption (at midpoint)

The midpoint results of the BoP Food in EU and of an average EU citizen in 2015 are presented in Table 3. As explained in the methodology section, the system boundary includes the entire food production and consumption, namely: primary production, manufacturing, distribution, use, and end of life. A comparison with the results without accounting for the recycling and reuse in the end of life stage are presented in SM (Table S12).

**Table 3:** Midpoint results of the BoP Food, representing the consumption of representative food products in Europe (Total EU-28) and of an average EU-28 citizen in 2015. The characterization have been performed using EF 2.0 method.

Impact category	Unit	Total EU-28	Average EU-28 citizen
Climate change	kg CO_2_ eq	1.38E+12	2.72E+03
Ozone depletion	kg CFC-11 eq	1.78E+06	3.51E-03
Human toxicity, non-cancer	CTUh	8.19E+04	1.61E-03
Human toxicity, cancer	CTUh	1.54E+04	3.03E-05
Particulate matter	Disease incidence	1.44E+05	2.83E-04
Ionising radiation	kBq U^235^ eq	2.72E+10	5.36E+01
Photochemical ozone formation	kg NMVOC eq	2.34E+09	4.60E+00
Acidification	molc H^+^ eq	1.97E+10	3.87E+01
Terrestrial eutrophication	molc N eq	8.37E+10	1.65E+02
Freshwater eutrophication	kg P eq	3.51E+08	6.90E-01
Marine eutrophication	kg N eq	8.91E+09	1.75E+01
Ecotoxicity freshwater	CTUe	3.68E+12	7.25E+03
Land use	Pt	1.33E+14	2.62E+05
Water use	m^3^ water eq	2.59E+12	5.09E+03
Resource use, fossils	MJ	7.94E+12	1.56E+04
Resource use, mineral and metals	kg Sb eq	1.24E+06	2.45E-03

In the majority of impact categories, the highest impact is due to meat consumption, followed by other animal-based products (milk, cheese, butter and eggs, see Table 4). The high impact of animal-based products is mainly due to the production of animal feeds (for further details, see the elementary flow contribution in SM, Table S10). However, regarding ozone depletion, about half of the impact is due to CFC-113, a refrigerant used in the storage and transportation of the cold or frozen products, i.e. meat, fish, dairy products, tofu and potatoes. Consumption of cod, representative product of wild catch, can also be identified as hotspot in photochemical ozone formation potential, due to the high fuel consumption during fishing.

**Table 4:**
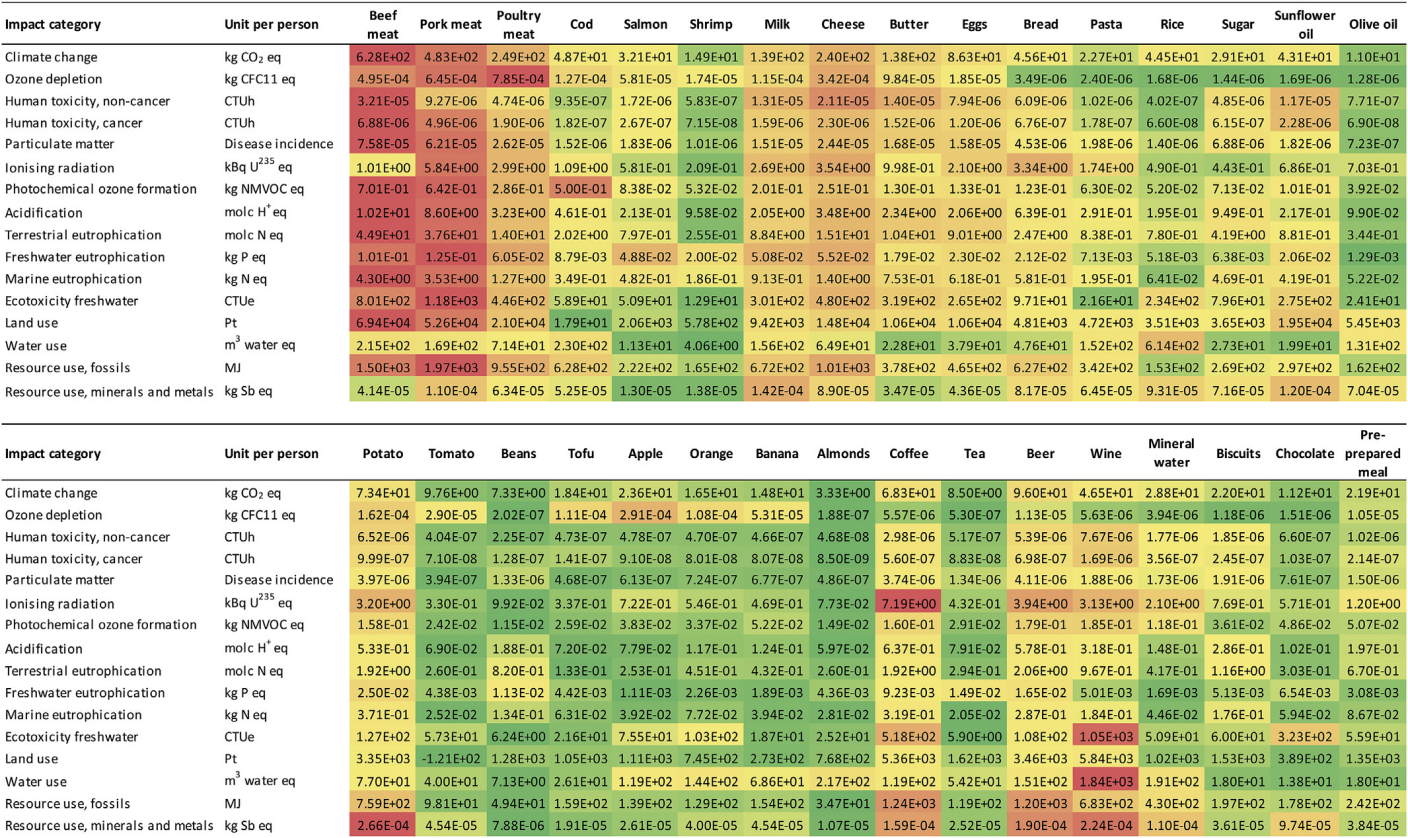
Midpoint results of the BoP Food, expressed as impacts of an average EU-28 citizen in 2015. The importance of the different food products is shown through the color code from red = highest impacts to green = lowest impacts.

Regarding plant-based products, potato and beer show the highest contribution to most of the impact categories due to high consumption amounts (beer: 70 L per year, potatoes: 68.5 kg per year), together with wine and coffee (Table 4). Wine consumption occurs as a hotspot in freshwater ecotoxicity, water use, and mineral and metal resource use. Coffee can be identified as a hotspot in many impact categories, despite the low consumption amount (3.3 kg per capita per year).

In order to complete the assessment, not only the overall impact is assessed (namely considering the amount consumed), but also the impact per kg. Meat has high impacts both per kg food and per the overall mass consumed. Other food products can be identified as hotspots when assessing impacts per kg product (see impacts per kg product in SM, Table S11). Tea, almonds and shrimps has low consumption amounts (0.6, 0.6 and 1.5 kg per capita per year, respectively), thus having low share of impacts in the food consumption of an average citizen. However, they occur as hotspots in many impact categories per kg product. For example, almond has the highest water use per kg of the representative products included in the study, while tea reports the highest mineral and metal resource use due to the high amount of packaging per kg product. In contrary, beer is identified as hotspot when the consumption amount is taken into account, while its impact per kg product is low.

### 3.2 Impact on biodiversity due to EU food consumption (at endpoint)

The endpoint results are presented in the next sections: firstly, focusing on analysing all drivers of biodiversity loss and their relative importance comparing the results of Recipe 2008 and 2016 applied to the BoP Food; secondly, focusing the comparison on the damage due to land use and land use changes. The endpoint results without considering the recycling and reuse in the end of life phase are presented in the supporting material.

#### 3.2.1 Impacts on biodiversity due to different drivers

**Fig. 2:**
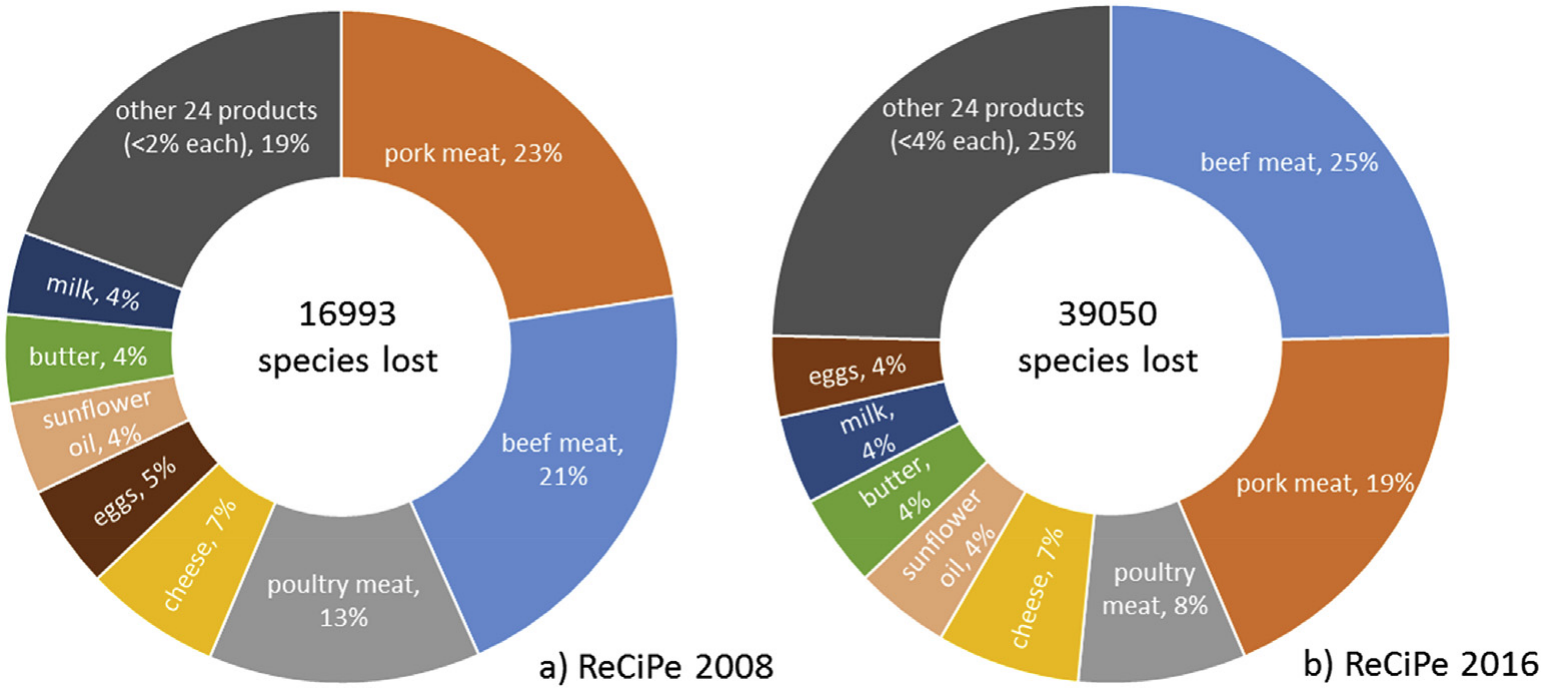
Overview of the relative contribution of each food product in the BoP Food to damage on biodiversity in 2015, based on ReCiPe 2008 and 2016. Note that the number of species lost is to be considered for comparative purposes.

Applying ReCiPe 2008 and 2016 to the BoP Food (as detailed above in section 2.1 and in SM) led to different results in absolute terms, although a similar pattern in the relative contribution of the impact categories at endpoint level is observed (Fig. 2). Notwithstanding these results are meant to be used on a comparative basis, according to the purposes of LCA practice, both absolute values are in line with the estimated magnitude of biodiversity decline (i.e. between 1000 and 10000 times the natural background extinction rate according to Chivian et al., 2008). Both methods highlight that the same eight products contribute to more than 75% of total damage to biodiversity, although with slight differences in the ranking. These differences arise as ReCiPe 2016 include updated damage pathways underpinning the impact assessment models for several indicators, which generate different characterization factors.

The methods indicate that a few impact categories contribute to more than 90% of the total damage caused by the BoP Food on ecosystem quality, in terms of species lost. In fact, land use and land transformation related impact categories and climate change play a leading role. In ReCiPe 2016, these categories are followed by terrestrial acidification, while in ReCiPe 2008 it does not represent a top-level driver of damage on ecosystems, contributing to less than 1% (see Table 5, Table 6).

**Table 5:** Endpoint results of the BoP Food, both for the total consumption in EU-28 and of an average EU-28 citizen, by means of ReCiPe 2008. The endpoint results are reported only for the Area of Protection “ecosystem quality”, which indicates the impacts on biodiversity.

Impact category at endpoint level (ReCiPe 2008)	Abbreviations	Unit	EU-28 consumption impacts	Per capita consumption impacts	% impact of EU consumption on biodiversity
Climate change	CC	species.yr	9.84E+03	1.94E-05	25.19%
Terrestrial acidification	AC	species.yr	9.18E+01	1.81E-07	0.24%
Freshwater eutrophication	FEU	species.yr	1.60E+01	3.15E-08	0.04%
Terrestrial ecotoxicity	ECOTOX-T	species.yr	1.92E+03	3.78E-06	4.92%
Freshwater ecotoxicity	ECOTOX-F	species.yr	1.82E+01	3.58E-08	0.05%
Marine ecotoxicity	ECOTOX-M	species.yr	6.63E-01	1.31E-09	0.00%
Agricultural land occupation a	ALO	species.yr	1.95E+04	3.85E-05	50.05%
Urban land occupation a	ULO	species.yr	1.06E+02	2.09E-07	0.27%
Natural land transformation a	NLT	species.yr	7.51E+03	1.48E-05	19.24%
**Overall impacts on biodiversity**	**-**	**species.yr**	**3.91E+04**	**7.69E-05**	**100.00%**

a Total land use- related results stand at 2.72E+04 species.yr (69.57%).

**Table 6:** Endpoint results of the BoP Food, both for the total consumption in EU-28 and of an average EU-28 citizen, by means of ReCiPe 2016. The endpoint results are reported only for the Area of Protection “ecosystem quality”, which indicates the impacts on biodiversity.

Impact category at endpoint level (ReCiPe 2016)	Abbreviations	Unit	EU-28 consumption impacts	Per capita consumption impacts	% impact of EU consumption on biodiversity
Global warming, terrestrial ecosystems	CC-T	species.yr	3.93E+03	7.73E-06	23.12%
Global warming, freshwater ecosystems	CC-F	species.yr	1.07E-01	2.11E-10	0.00%
Ozone formation, terrestrial ecosystems	POF-T	species.yr	2.20E+02	4.32E-07	1.29%
Terrestrial acidification	AC	species.yr	2.70E+03	5.31E-06	15.88%
Freshwater eutrophication	FEU	species.yr	3.15E+02	6.21E-07	1.86%
Terrestrial ecotoxicity	ECOTOX-T	species.yr	2.22E+01	4.37E-08	0.13%
Freshwater ecotoxicity	ECOTOX-F	species.yr	4.05E+00	7.97E-09	0.02%
Marine ecotoxicity	ECOTOX-M	species.yr	4.07E-01	8.01E-10	0.00%
Land use a	LU	species.yr	8.86E+03	1.74E-05	52.14%
Water consumption, terrestrial ecosystems	WU-T	species.yr	9.42E+02	1.85E-06	5.55%
Water consumption, aquatic ecosystems	WU-F	species.yr	4.22E-02	8.30E-11	0.00%
**Overall impacts on biodiversity**	**-**	**species.yr**	**1.70E+04**	**3.34E-05**	**100.00%**

a It includes both land occupation and transformation.

The general pattern of results is in line with recently published results regarding LCAs of a food retailer company (Wolff et al., 2017), although their focus was on a global scale.

**Fig. 3:**
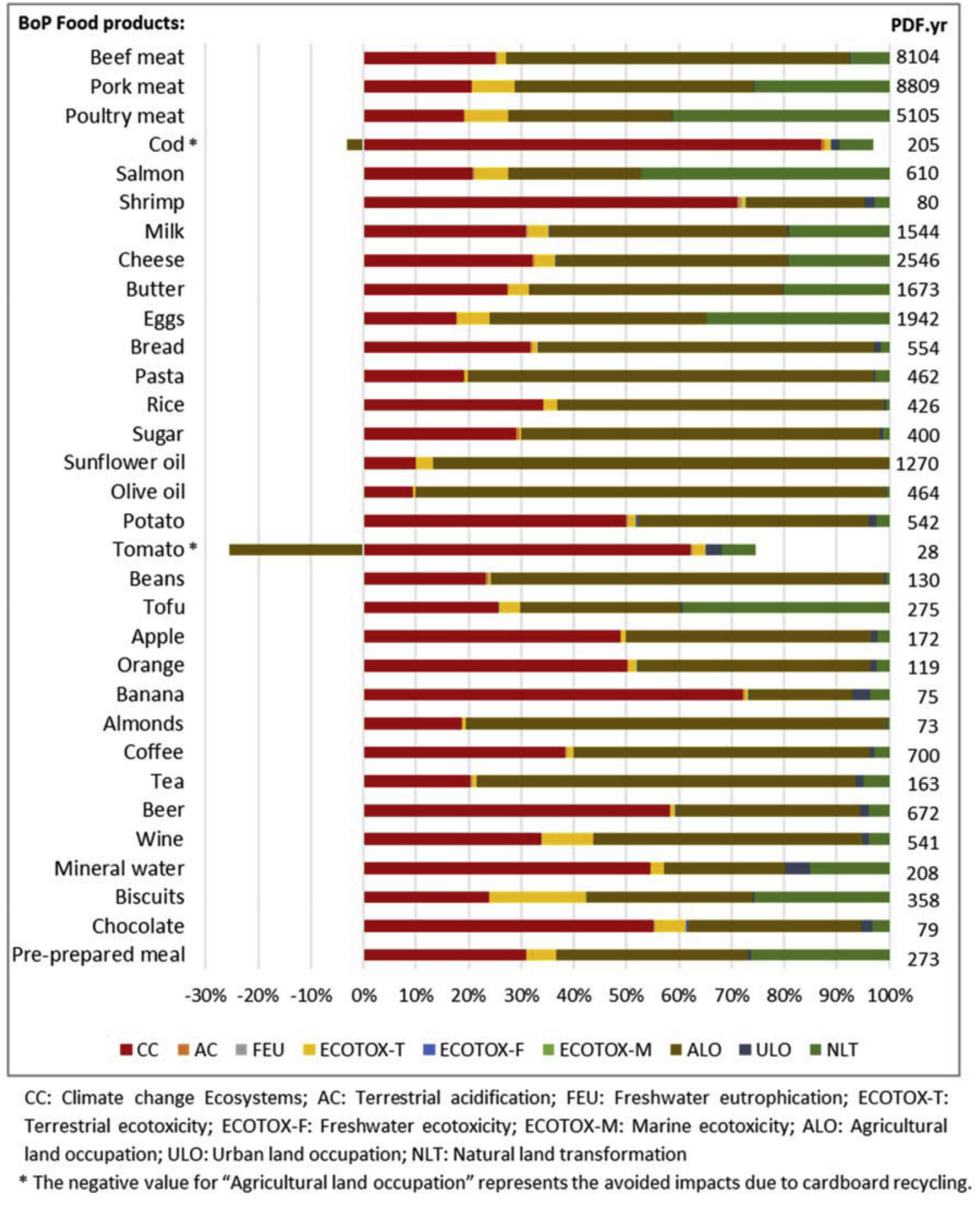
Contribution of the midpoint impact categories to the impacts on biodiversity by product, applying ReCiPe 2008. Absolute results in terms of species lost per year are reported on top of each impact category.

**Fig. 4:**
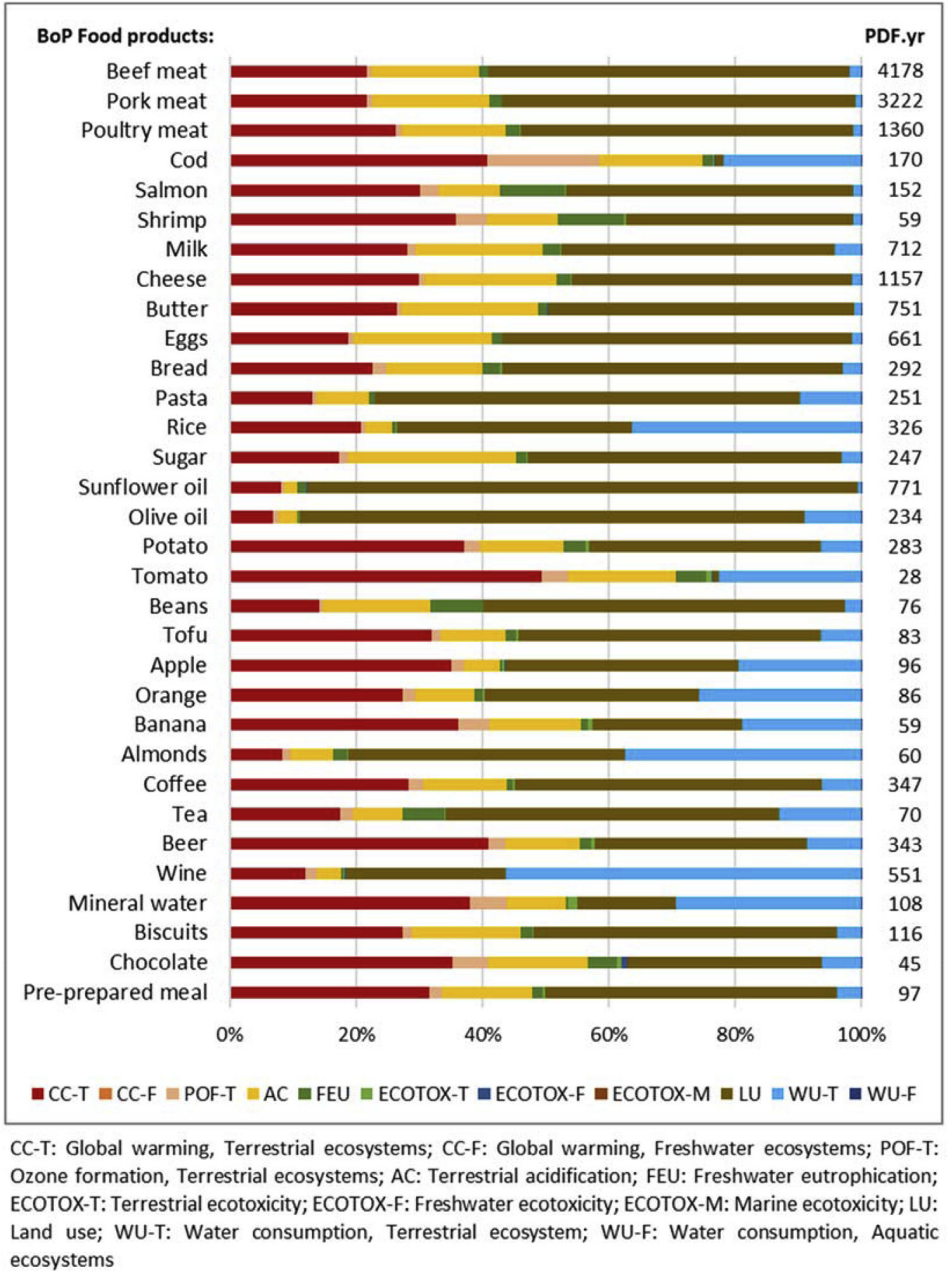
Contribution of the midpoint impact categories to the impacts on biodiversity by product, applying ReCiPe 2016. Absolute results in terms of species lost per year are reported on top of each impact category.

Also at product level, the impact categories mentioned above play a leading role in determining species loss (Fig. 3, Fig. 4). Specifically, in both methods, land use related impact categories contribute to more than 50% of damage in at least 11 products, while climate change contribution is generally higher than 25%.

Loss of species is mainly driven by meat, specifically pork and beef, which together contribute to 43% of total species loss over a year according to both ReCiPe 2008 and 2016. The reason behind the relevant role of meat is twofold, namely the intensity of the impacts of a certain food type per kg and its amount consumed at EU-28 level. For instance, beef meat has an annual consumption of 15.2 kg per person in 2015, which is lower compared to other meat products, such as pork. However, beef is the meat-product food type with the greatest environmental burden since it has the highest environmental impact per kg. Conversely, pork meat presents a lower environmental burden compared to beef, but this is counterbalanced by higher per-capita consumption, resulting in a high share of the overall impact (Notarnicola et al., 2017). The 13 new products identified in this study contribute all together to about 11% of total damage on ecosystem quality according to both methods, with eggs showing the highest share of damage (5% and 4% in ReCiPe 2008 and 2016, respectively).

For some products, namely cod and tomatoes, both methods provide negative results associated with agricultural land occupation. This is more evident in the results of ReCiPe 2008, while in ReCiPe 2016 it is offset by the high impact of arable land occupation (higher CFs) (Fig. 3, Fig. 4). Given the way in which the end of life of the products is modelled, the recycling of materials is leading to a benefit (an impact with a negative sign) in terms of avoided impact related to the virgin production. In the specific case, negative results are due to the contribution of the flow related to “occupation, forest, intensive", which is avoided when recycling paper-based materials. In fact, by avoiding the land use impacts underpinning the production of novel cardboard packaging, the system gains a benefit on the overall environmental damage due to land use. This benefit is not evidently shown in other products, such as beer, pasta and cheese that include a recycling phase in the end of life of their cardboard packages. In fact, in their inventory the occupation of arable land for cultivation dominates over the forested area occupation, compared to cod (wild fish, i.e. no land use in beginning of the life cycle) and tomatoes (which have a high yield, thus do not need large areas for cultivation). This result shows how sensible is the assumption of the end of life modelling. For example, when considering the system without recycling, no benefits occur (see Figure S1-S2 in the SM).

Although the ranking of endpoint categories is similar in both ReCiPe 2008 and ReCiPe 2016, in some cases, the two methods do not converge on pointing out the same hotspots, namely at the level of which actions are to be taken to reduce the environmental burden. For instance, considering the life cycle of wine, ReCiPe 2008 identifies agricultural land occupation as the most contributing category, while by applying ReCiPe 2016 it would have to intervene on other features (e.g. water consumption) in order to reduce the biodiversity impacts due to the consumption of wine. In fact, ReCiPe 2016 includes a higher number of environmental interventions, namely it includes the damage pathway associated with water use, a crucial hotspot for rice, vineyards and almonds cultivation that in ReCiPe 2008 is not highlighted because this category was not addressed. The updated method also addresses the pathway for climate change leading to damages in freshwater ecosystems and the one for photochemical ozone formation into terrestrial ecosystems (Huijbregts et al., 2016). On the other hand, the two methods converge on identifying land use as the main hotspot for bread, pasta, olive oil, and sunflower oil.

#### 3.2.2 Impacts on biodiversity due to land use

According to both ReCiPe 2008 and 2016, meat products lead the environmental burden on biodiversity (Fig. 3, Fig. 4 respectively). Considering the total damage due to the three land use and transformation related categories in ReCiPe 2008, pork meat, followed by beef meat, represents the product with the highest share of impact on species richness (23%). For both products, the main underpinning cause is the transformation of natural land and its consequent occupation for agricultural purposes. The cultivation of barley and soybean, used as feed, represent the most contributing processes. By applying ReCiPe 2016, beef meat leads the impacts on biodiversity, followed by pork meat (27% and 20% respectively). These figures are associated with livestock grazing in pasture (49%) and the production of grass as feed (27% of total impact for beef meat). The main elementary flow behind this impact is the occupation of arable land, which covers almost 100% of the impact.

The endpoint results generally show that land use for agricultural purposes (i.e. arable land occupation, distantly followed by intensive forestry) is the predominant contributor to impacts on biodiversity, greatly overcoming the impacts from land transformation (see contribution analysis in SM, Tables S13-S14).

Concerning the characterization framework developed by Chaudhary et al. (2015), two versions of the method were implemented in SimaPro v.8.5 (Pré-Sustainability, 2018). In a first version, the six land use type flows according to the original method (i.e. arable, permanent crops, pasture and meadow, urban, forest extensive and intensive), both occupation and transformation, were mapped. Then, in a second version of the method all the sub-types of land use (i.e. second and third level according to Koellner et al., 2013) were mapped by using the CF of the highest level, in order to broaden the coverage of the inventory.

By applying the first version of the method, all the four sets of CFs highlightes that pork meat, followed by beef and poultry meat, represents the food product that mostly affects biodiversity, in terms of both regional loss and global extinctions (see SM, Table S15). The result is in line with ReCiPe 2008, although with slightly higher figures. The impact does not depend on the country, since the “global average” CFs used are by definition not regionalized. The result mainly depends on the use of agricultural land for cultivating crops for feed (e.g. soybean). Shrimps appear to be the least impacting food product, with an apparent benefit (negative value of around −3%). On the one hand, this result is due to a benefit from the recycle of cardboard packaging, as underlined in the above-mentioned case of cod and tomatoes. However, this negative result is also associated with a limitation of the method itself. In fact, although at the inventory level the characterization by Chaudhary et al. (2015) covers 91% of the occupied area of the BoP Food inventory, the flow coverage of this method is limited to a reduced number of flows, namely 14 out of 138 flows included in the BoP Food inventory are characterized. The method characterizes the flows at the highest level of CF (e.g. "arable" class, without covering more detailed features, such as intensive, extensive, irrigated, etc.). In this way, important impacts are missing, e.g. "transformation, to arable, non irrigated, intensive", which plays a role in balancing the flow related to transformation from arable in shrimp-aquaculture phase, remains uncharacterized.

The situation changes when adopting the version mapped more in details, namely mapping the lower levels of land use type (see SM, Table S16). In fact, shrimps turn to have an environmental impact on biodiversity, although minimal with respect to other products, and the benefit from cod and tomato packaging recycling emerges in accordance with Recipe 2008. However, it has to be considered that the remapping of flows at lower levels may lead to potential under- or overestimation of impacts. For example “occupation, forest, intensive, short-cycle” was mapped with the same CF as “occupation, forest, intensive”, which has a higher impact; similarly, “occupation, arable, irrigated, intensive” was mapped with the CF of “occupation, arable”, which has a lower environmental impact.

## 4 Discussion

**Fig. 5:**
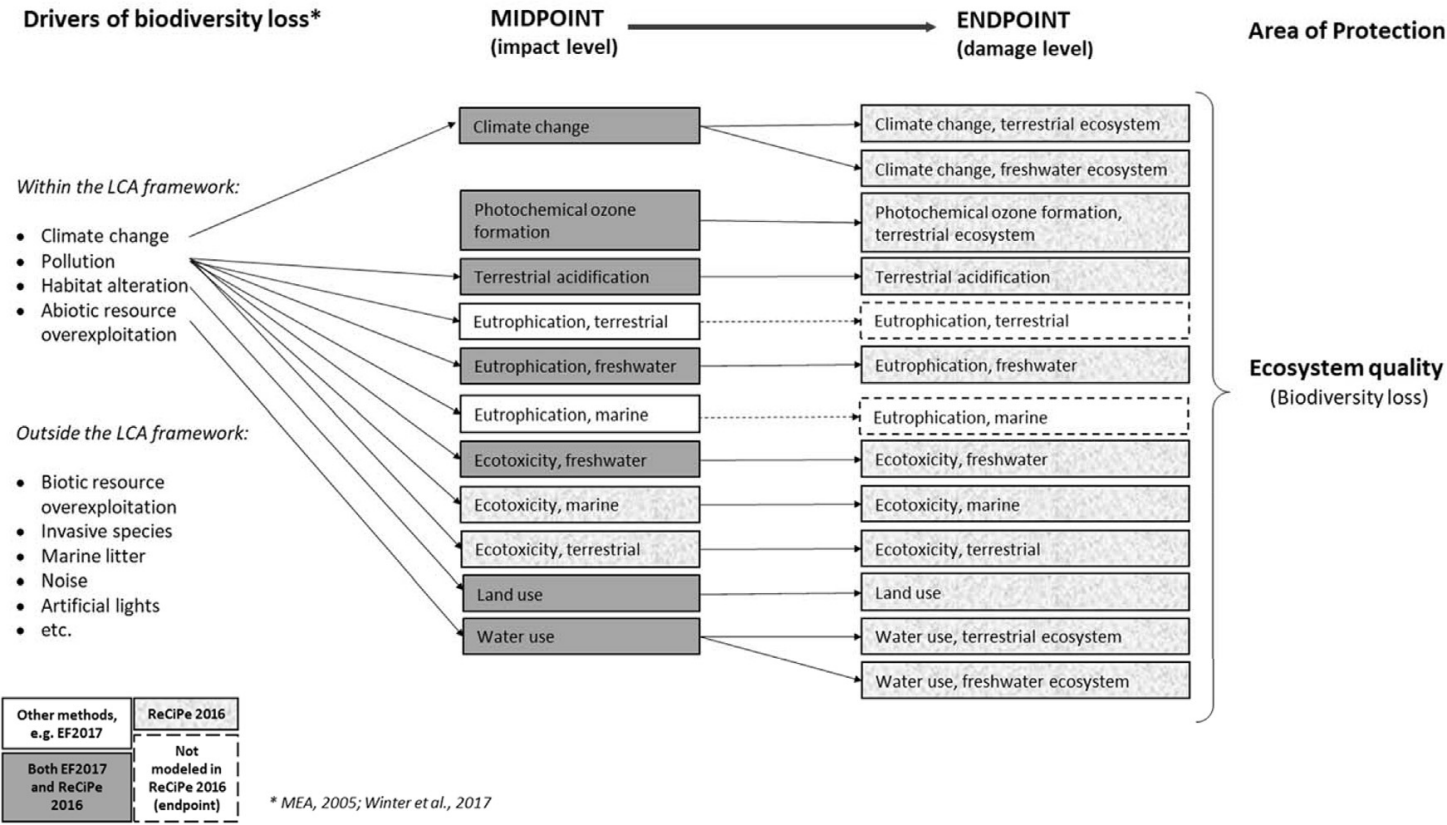
Overview of the impact drivers leading to biodiversity loss and their status in the LCIA framework, according to specific methods (adapted from Winter et al., 2017).

The midpoint results show that the highest environmental impacts - in the majority of the impact categories- are due to the consumption of meat and dairy products. The same products have the highest impact also in the study of Notarnicola et al. (2017). In fact, these products have high environmental impact per kg product, but also a high overall consumed amount. This is reflected at endpoint level, where ecosystem quality and biodiversity are mainly affected by the consumption of pork and beef meat. On the contrary, the products that were selected due to their expected high environmental impacts (almonds, shrimps, tea) are not showing relevant impacts in the total food consumption due to their relatively low consumption amount. At endpoint level, both ReCiPe methods converge on pointing out land use and climate change as the indicators leading to the highest damage on ecosystem quality and biodiversity, as in literature (MEA, 2005; Barnosky et al., 2011). However, these are only part of the bunch of impacts leading to biodiversity loss. In fact, in LCA today only a few drivers of biodiversity loss are modelled and addressed as impact categories. Specifically, four out of five drivers of biodiversity loss identified by the Millennium Ecosystem Assessment (i.e. habitat alteration, climate change, pollution, resource overconsumption and biotic exchange in terms of spread of invasive species) are represented in the existing midpoint/endpoint impact categories (Fig. 5). Most of the missing drivers are linked to agricultural practice and international trade, such as the spread of invasive and alien species (EC, 2017b). Additionally, some pressures (e.g. overexploitation of biotic resources as overfishing, etc.), some compartments or habitat (e.g. coastal system for shrimp aquaculture, seafloor impacts due to fishing, etc.), or species (e.g. pollinators) are not yet captured in the impact assessment modelling, thus leading to uncertain or limited results. Refinements are therefore needed.

Over time, a number of studies tried to cover the missing drivers in the LCA framework. For example, Hanafiah et al. (2013) and Emanuelsson et al. (2014) assessed respectively the impacts due to the spread of invasive species and overfishing (see Winter et al., 2017 for more details). Crenna et al. (2018) identified the key aspects for developing a characterization framework able to assess the impacts on the availability of biotic resources. Winter et al. (2018) developed a comprehensive impact assessment framework for quantifying impacts of products on biodiversity, by addressing the main existing limitations. However, these models are available only in case studies and are not yet operational in the common LCA practice. Other critical impact drivers are still missing in the LCIA framework, including noise, artificial lights and thermal pollution (as highlighted by Winter et al., 2017) and the international trade, recently identified as a key point of biodiversity loss to address in LCIA. In fact, the international import-export system of the current globalized economies is increasing the rate of habitat degradation and species loss in those areas where products have their origin (Lenzen, 2012; Moran et al., 2016; Moran and Kanemoto, 2017).

More recent research for specific impact categories, e.g. land use, has been performed (Chaudhary et al., 2015, 2016; Curran et al., 2016; Teixeira et al., 2016). Land use represents the focus of the majority of modelling efforts towards the inclusion of biodiversity in the LCA framework. However, some aspects are still under improvement or not yet covered. In fact, while spatial differentiation of impacts is addressed by SAR models underpinning CFs in ReCiPe 2016 and Chaudhary et al. (2015), the role of habitat fragmentation is not assessed within the recommended UNEP/SETAC LCIA guidelines (Larrey-Lassalle et al., 2018).

Ecosystems are heterogeneous and their quality is highly complex to monitor and assess. In fact, different biomes hold different biodiversity potential, in terms of species richness, abundance and community composition (MEA, 2005). In ReCiPe 2008 and its updated 2016 version, the assessment of loss in biodiversity, which underpins the good functioning of ecosystems, is defined simply in terms of loss of species richness. This entails accepting the basic assumption that species diversity fairly represents the total quality of ecosystems (Goedkoop et al., 2009). The use of species richness as metric in the LCIA context is driven by the fact that loss of representative species groups is seen as indicative for a general biodiversity decline and the potential loss of resilience (Callesen, 2016). In fact, the extinction of a species on a global scale is an irreversible loss of biological information, which may also affect other species and the functioning of the ecosystems. However, this metric is not properly adequate for several reasons. For instance, PDF tends to be estimated for vertebrates, which represent only 2% of all described species, and it does not take into account changes in species abundance, community composition and distribution of species (Mace et al., 2014; Winter et al., 2018).

Generally, the taxonomic coverage of the existing LCIA models is limited and the choice of representative species is different for each stressor, namely only a small number of specific taxa for individual impact pathways are used to develop impact factors (Woods et al., 2017). A few vertebrates and invertebrate taxa are addressed for land use related impacts on biodiversity (i.e. mammals, birds, amphibians, and reptiles in Chaudhary et al., 2015; additionally snails, spiders, carabids, butterflies, wild bees, and grasshoppers in the SALCA model by Jeanneret et al., 2008). Whereas, vascular plant species are adopted in other models, e.g. for acidification (e.g. ReCiPe underpinning models developed by Van Zelm et al., 2007). However, these selected species groups are poorly linked to the EU statistics. In fact, in the current EU Strategies for halting biodiversity decline (EC, 2011; EU, 2013), key indicators are the population trends of common birds and grassland butterflies (EEA, 2018).

Additionally all species are generally considered equivalent in the current LCIA models, without taking into account their distribution on local, regional and global scale (Woods et al., 2017) and without integrating important ecological aspects, such as vulnerability as identified by the IUCN Red List (IUCN, 2018) and the spatial-explicitness of impacts on biodiversity. These factors have been barely addressed in LCA so far (e.g. Chaudhary et al., 2015). Nevertheless, they could be key for the definition of species loss on different scales, stressing on the concrete context of localized ecosystems. Hence, it is crucial to ensure a better comparability between environmental impacts, especially when the LCIA comes to support decision making for regional purposes.

In the specific context of land use driven damage to ecosystem quality, it would be interesting to display results per hectare as well. In fact, for many indicators measuring biodiversity impacts, per hectare values may be more informative, being the impact dependent on where it arises. Although per hectare indicators still do not take into account spatial heterogeneity, it could capture the pressure on ecosystems better than a mass-based indicator.

Beyond the LCA field, both the public and the private sectors have made efforts to address environmental sustainability challenges, towards the definition of targets for safeguarding biodiversity. Inspired by the concept of planetary boundary (Rockström et al., 2009; Steffen et al., 2015; Mace et al., 2014) several One Planet Approach (OPA) methodologies have been developed. A recent example is the holistic and integrative approach proposed by WWF and IUCN in association with a consulting company (Sabag Muñoz and Gladek, 2017). It aims at identifying sustainable targets in line with the Earth's carrying capacity through the definition of impact areas as priority for companies operating in the agro-food sector, thus including biodiversity loss (WWF, 2018). However, the definition of the ecological boundaries for biodiversity, as for example referred to in Wolff et al. (2017), still needs to be clarified. Biodiversity conservation targeted solutions need to be consistently referred to the carrying capacity of the ecosystems.

## 5 Conclusions

This study focuses on the assessment of biodiversity impacts due to food consumption in EU.

Firstly, we updated the work done by Notarnicola et al. (2017) both temporally (from year 2010 to year 2015) and in terms of total number of food products. The aim was to better account for EU food consumption, through a more complete selection of representative food products (e.g. tomatoes and eggs), also including emerging food trends (e.g. vegetarian food), as well as food types associated with high environmental impacts (e.g. almonds, rice, tea, chocolate).

The results of the LCA study carried out to assess the environmental impacts of the average EU food consumption in 2015 show that the products that affect the most the environment across all the impact categories are meat and dairy products, as in the study of Notarnicola et al. (2017). By identifying the main hotspots, such as freshwater ecotoxicity and water use related to wine, ionising radiation related to coffee and photochemical ozone formation related to cod, the results may help the assessment of the environmental impacts of the average EU food consumption towards a more responsible and sustainable production-consumption system.

This study also represents a preliminary LCA-oriented exercise on the evaluation of the role of EU food consumption system in biodiversity decline, which is a hot topic in both the ecological, environmental, and economic context. Although the endpoint modelling is still considered highly uncertain (Kägi et al., 2016), it may highlight aspects to be further considered. In fact, the endpoint modelling represents a simplified damage system built on a science-based aggregation of the impact categories, which facilitates the comparison and interpretation of impacts to society values (UNEP, 2000). For this reason, it is suggested to use the most up-to-date methods, which result from the current scientific state of the art, thus dealing with uncertainty in a better and more solid way.

Furthermore, the endpoint analysis may help to identify the main hotspots of impact on which intervene in order to reduce the environmental burden and meet the sustainable development goals set at international level (e.g. SDG 14 and 15 on the conservation of life below water and on land respectively). In fact, all the applied endpoint methods converge on identifying animal-based products, such as beef and pork meat, as the main contributors to species loss. This is mainly due to the transformation of natural land and its consequent occupation for agricultural purposes, such as cultivation of feed-plants. However, in some cases, the two methods do not converge on pointing out the same hotspots of impacts, due to difference in the coverage of the impact categories and simplifications in the underpinning modelling of the impact pathways.

Finally, the evidence of the increasing biodiversity decline on a global scale indicates that the LCA accounting system should be further explored, especially for what concerns refining ecotoxicity and land use impact modelling and including biotic resource overexploitation and invasive species among the modelled impacts. In fact, the impact modelling underpinning ecotoxicity should be expanded to different taxonomic groups (e.g. pollinators, Crenna et al., 2017) and characterization factors refined (Saouter et al., 2017, 2018), as well as the different intensity of land management addressed (as recently highlighted in Chaudhary and Brooks, 2018).

Biodiversity impact assessment is very complex and affected by many context-related, spatial and temporal differences. However, notwithstanding the limitations and uncertainties associated to the assessment of biodiversity impacts at macro scale and the possibility of giving an absolute number, the present study is clearly identifying hotspots that should be considered in terms of their implications for policy-making. Current European diet is affecting biodiversity both in Europe and on a global scale. Meat and dairy consumption are the main drivers of impacts, suggesting that a balanced and healthy diet may bring both environmental and social benefits.
