# Supplementary material for: Biodiversity impacts due to food consumption in Europe
Source: J Clean Prod. 2019 Aug 1;227:378–91. doi: 10.1016/j.jclepro.2019.04.054 (PMC6559135; doi:10.1016/j.jclepro.2019.04.054)
Supplement: Multimedia component 1 [file mmc1.docx]

**Supplementary material**

**Biodiversity impacts due to food consumption in Europe**

Crenna, E.^1^, Sinkko, T.^1^, Sala, S.^1^

^1^ European Commission, Joint Research Centre (JRC), Ispra, Italy

**Contents**

[1. Life cycle inventory data of selected products 2](#_Toc519161998)

[1.1. Agricultural stage 2](#_Toc519161999)

[1.2. Processing 6](#_Toc519162000)

[1.3. Packaging 7](#_Toc519162001)

[1.4. Logistics 8](#_Toc519162002)

[1.5. Use phase 10](#_Toc519162003)

[1.6. End-of-life 10](#_Toc519162004)

[2. Additional information on the results 11](#_Toc519162005)

[2.1. Characterized results of EU food consumption at midpoint 11](#_Toc519162006)

[2.1.1. Comparison of results: system with vs system without reuse and recycling 14](#_Toc519162007)

[2.1.2. Flow contribution to the overall environmental impact 11](#_Toc519162008)

[2.1.3. Results per kg food product 12](#_Toc519162009)

[2.2. Characterized results of EU food consumption at endpoint: impacts on biodiversity 14](#_Toc519162010)

[2.2.1. Flow contribution to biodiversity loss 14](#_Toc519162011)

[2.2.2. Biodiversity impact assessment of the BoP Food, without accounting for reuse and recycling 16](#_Toc519162012)

[References 23](#_Toc519162013)

# **Life cycle inventory data of selected products**

The life cycle inventory data of the 13 products selected in this study to complement the work done by Notarnicola et al. (2017) and Castellani et al. (2017) is reported below, by life cycle stage. In addition, inventory data of the original work is presented.

## **Agricultural stage**

Table S1 reports data regarding cultivated crops per cultivated hectare. The emissions from the combustion of diesel in the agricultural machinery have not been reported in this table, but are considered in the inventory according to the Agri-footprint database process ‘Energy, from diesel burned in machinery’ (Blonk Consultants, 2014). Table S1 includes only crops modelled in this study. Cultivation of other plant-based food (beans), or raw materials used (e.g. soya beans for tofu), are based on Agri-footprint database (Blonk Consultants, 2014), and thus not reported in Table S1. Table S2 includes the inventory data of agricultural phases already reported in Notarnicola et al. (2017) and Castellani et al. (2018).

**Table S1**. Inventories of the agricultural phase of the products selected in this study (per cultivated ha per year).

|  | **Unit** | **Banana^1)^** | **Tomato^3)^** | **Rice^4)^** | **Cocoa^6)^** | **Almonds^7)^** | **Tea^8)^** |
| --- | --- | --- | --- | --- | --- | --- | --- |
| Products | t | 32.8 | 166.7 | 7.0 | 25.5 | 3.3 | 1.7 |
| Co-products (total weight) | t | 0 | 0 | 8.4 | 0 | 0 | 0 |
| **Inputs** | | | | | | | |
| N fertiliser | kg | 295 | 798 | 130 | 0 | 180 | 306 |
| P fertiliser | kg | 38 | 506 | 18 | 818 | 100 | 728 |
| K fertiliser | kg | 131 | 1562 | 135 | 670 | 200 | 128 |
| Lime | kg | 22 | 0 | 0 | 0 | 0 | 0 |
| Pesticides (total weight) | kg | 15 | 46.5 | 16.7 | 210 | 8.2 | < 0.1 |
| Irrigation water | m^3^ | 3083^2)^ | 4748 | 4087^5)^ | 131 | 4650 | 136 |
| Diesel | kg | 108 | 0 | 95.5 | 0 | 539 | 14.3 |
| Heat | MJ | 0 | 0 | 4280 | 0 | 0 | 0 |
| Electricity | kWh | 10 | 6485 | 51 | 0 | 803 | 0 |
| **Outputs** | | | | | | | |
| ***Emissions to air*** | | | | | | | |
| N_2_O from fertilisers | kg | 6.1 | 16.6 | 2.7 | 0 | 3.7 | 6.4 |
| NH_3_ from fertilisers | kg | 35.9 | 97.0 | 15.8 | 0 | 21.9 | 37.2 |
| CO_2_ from fertilisers | kg | 9.5 | 0 | 80.1 | 0 | 0 | 0 |
| CH_4_ from field | kg | 0 | 0 | 338 | 0 | 0 | 0 |
| ***Emissions to water*** | | | | | | | |
| NO_3_ from fertilisers | kg | 89 | 239 | 39 | < 0.1 | 54 | 92 |
| P from fertilisers | kg | 2 | 25 | 1 | < 0.1 | 5 | 36 |
| ***Emissions to soil*** | | | | | | | |
| Bispyribac-sodium | kg | 0 | 0 | 0.3 | 0 | 0 | 0 |
| Captan | kg | 0 | 0 | 0 | 0 | 4.1 | 0 |
| Chlorpyrifos | kg | 0 | 3.8 | 0.2 | 9.1 | 0 | 0 |
| Diuron | kg | 0 | 0 | 0.1 | 0 | 0 | 0 |
| Glyphosate | kg | 2.8 | 0 | 0.8 | 0 | 0 | 0 |
| Mancozeb | kg | 6.2 | 28.5 | 0 | 142 | 0 | < 0.1 |
| Pretilchlor | kg | 0 | 0 | 4.6 | 0 | 0 | 0 |

^1)^ Data based on cultivation in Ecuador (Iriarte et al., 2014)

^2)^ Mekonnen and Hoekstra, 2011

^3)^ Data based on cultivation in greenhouse in Spain (Torrellas et al., 2012)

^4)^ Data based on cultivation in Italy (Blengini and Busto, 2009)

^5)^ Takes into account water uptake of the rice and available rainwater in the area. Although rice is usually cultivated in flooded field, but part of this water can be captured and reused downstream (Chapagain and Hoekstra, 2010).

^6)^ Data based on cocoa cultivation in Ghana (Ntiamoh and Afrane, 2008)

^7)^ Data based on almond cultivation in Greece (Bartzas et al., 2017)

^8^) Data based on average of Kenya, India and Indonesia (Jefferies et al., 2012)

**Table S2**. Inventories of the agricultural phase of original products (per cultivated ha per year) (already published in Notarnicola et al. (2017) and Castellani et al. (2017)).

|  | **Unit** | **Apple** | **Orange** | **Potato** | **Barley** | **Wheat** | **Coffee** | **Sugar beet** | **Sunflower seeds** | **Grape** |
| --- | --- | --- | --- | --- | --- | --- | --- | --- | --- | --- |
| Products | t | 31.4 | 25.0 | 41.6 | 5.7 | 7.1 | 9.0 | 58.9 | 1.3 | 1.6 |
| Co-products (total weight) | t | - | - | - | 4.0 | 4.0 | - | - | - | - |
| **Inputs** | | | | | | | | | | |
| N fertiliser | kg | 62 | 240 | 100 | 145 | 149 | 238 | 150 | 57 | 4 |
| P fertiliser | kg | 4 | 100 | 101 | 10 | 19 | 26 | 40 | 50 | 2 |
| K fertiliser | kg | 47 | 180 | 131 | 14 | 17 | 233 | 140 | 21 | 9 |
| Lime | kg | 52 | 0 | 365 | 329 | 327 | 1 057 | 291 | 400 | 0 |
| Compost | kg | 0 | 0 | 0 | 0 | 0 | 0 | 0 | 0 | 150 |
| Pesticides (total weight) | kg | 10.5 | 15.1 | 9.9 | 4.7 | 4.7 | 15.1 | 10.5 | 3.9 | 13.5 |
| Irrigation water | m^3^ | 3 000 | 4 000 | 351 | 0 | 0 | 0 | 186 | 33 | 5 |
| Diesel | kg | 231.7 | 250 | 244 | 131.2 | 138.5 | 161 | 164.5 | 92.6 | 33.1 |
| Electricity | kWh | 952 | 3 200 | 1 446 | 0 | 0 | 0 | 0 | 305 | 12 |
| **Outputs** | | | | | | | | | | |
| ***Emissions to air*** | | | | | | | | | | |
| N_2_O from fertilisers | kg | 1.3 | 5.0 | 5.0 | 5.4 | 5.4 | 5.0 | 11.3 | 1.8 | 0.2 |
| NH_3_ from fertilisers | kg | 7.5 | 29.1 | 43.4 | 43.8 | 42.4 | 28.9 | 107.3 | 13.8 | 0.4 |
| CO_2_ from fertilisers | kg | 43.3 | 23.5 | 204.7 | 234.1 | 235.8 | 669.4 | 202.4 | 189.0 | 1.8 |
| ***Emissions to water*** | | | | | | | | | | |
| NO_3_ from fertilisers | kg | 82.4 | 318.9 | 304.0 | 336 | 331.5 | 316.2 | 686.5 | 113.3 | 17.9 |
| P from fertilisers | kg | 0.1 | 2.2 | 3.5 | 1.3 | 1.3 | 0.6 | 2.2 | 1.2 | < 0.1 |
| ***Emissions to soil*** | | | | | | | |  |  |  |
| Azoxystrobin | kg |  |  |  | 0.1 | 0.1 |  |  |  |  |
| Captan | kg |  |  |  |  |  | 1.5 |  |  |  |
| Carbaryl | kg |  |  |  |  |  | 1.2 |  |  |  |
| Carboxin | kg |  |  |  |  |  |  |  | 0.5 |  |
| Chloridazon | kg |  |  |  |  |  |  | 0.5 |  |  |
| Chlorpyrifos | kg | 0.8 | 1.2 |  |  |  | 1.2 |  | 0.1 |  |
| Copper | kg |  |  |  |  |  | < 0.1 |  |  | 3.0 |
| Dimethoate | kg |  |  | 0.2 |  |  |  |  |  |  |
| Diquat | kg |  |  | 0.3 |  |  |  |  | 0.1 |  |
| Epoxiconazole | kg |  |  |  |  |  |  | 0.1 |  |  |
| Ethephon | kg |  |  |  | 0.1 | 0.1 |  |  |  |  |
| Ethofumesate | kg |  |  |  |  |  |  | 0.5 |  |  |
| Fluazinam | kg |  |  |  |  |  |  |  | 0.4 |  |
| Fosetyl-aluminium | kg |  | 0.5 |  |  |  |  |  |  | 3.0 |
| Glyphosate | kg | 0.7 | 4.0 |  | 0.3 | 0.3 | 2.0 | 0.5 |  | 0.7 |
| Mancozeb | kg | 2.0 | 0.5 | 4.8 |  |  |  |  |  |  |
| Mcpa – sodium salt | kg |  |  |  | 0.3 | 0.3 |  |  |  |  |
| Methomyl | kg |  |  | 0.1 |  |  |  |  |  |  |
| Mineral oil | kg | 1.6 | 1.2 | 0.3 |  |  |  |  |  |  |
| Pencycuron | kg |  |  |  | 0.3 | 0.3 |  |  |  |  |
| Phenmedipham | kg |  |  |  |  |  |  | 0.7 |  |  |
| Propiconazole | kg |  |  |  | 0.1 | 0.1 |  |  |  |  |
| Prosulfocarb | kg |  |  | 0.6 |  |  |  |  |  |  |
| Sulfur | kg | 2.1 |  |  |  |  |  | 0.5 |  |  |
| Tebuconazole | kg |  |  |  |  |  |  |  | 0.1 |  |
| Trinexapac-ethyl | kg |  |  |  | 0.1 | 0.1 |  |  |  |  |
| Unspecified pest. | kg |  |  |  |  |  |  |  |  | 2.0 |

Table S3 shows the inventories of the agricultural phase of animal-based products. Agricultural phase of egg production was based on Agri-footprint database (Blonk Consultants, 2014), thus inventory data of egg production is not presented here. In this study, no burdens were allocated to the co-products of fish filleting, because according to other studies (e.g. Ellingsen et al., 2009) fish filleting co-products have very small economic value, and they have not allocated anything to co-products. In addition, in fish feed process, no burdens are allocated to the raw materials derived from fish co-products.

**Table S3.** Inventories of the farming phase of animal-based products.

|  | **Unit** | **Salmon^1)^** | **Shrimps^2)^** | **Milk^3)^** | **Beef cattle for slaughter^3)^** | **Pigs for slaughter^3)^** | **Broilers for slaughter^3)^** |
| --- | --- | --- | --- | --- | --- | --- | --- |
| Products | kg | 1000 | 1 000 | 1 000 | 1 000 | 1 000 | 1 000 |
| Co-products (total weight) | kg | - | - | 25 | - | - | - |
| **Inputs** | | | | | | | |
| Compound feed | kg | 1 103 | 1 600 | 219 | 1 563 | - | 1 679 |
| Grass | kg | - | - | 1 364 | 21 376 | - | - |
| Grass silage | kg | - | - | - | 7 666 | - | - |
| Maize silage | kg | - | - | 717 | - | - | - |
| Mix of by-products | kg | - | - | 105 | - | - | - |
| Pig feed | kg | - | - | - | - | 2 057 | - |
| Water | m^3^ | - | - | 2 | 138 | 9 | 3 |
| Heat from gas | MJ | 0.1 | - | 57 | - | 99 | 1 179 |
| Diesel | kg | 12.8 | - | 130 | 130 | - | - |
| Electricity | kWh | 20.1 | 2 550 | 58 | 304 | 13 | 48 |
| **Outputs** | | | | | | | |
| N to water | kg | 41.1 | 66.0 | - | - | - | - |
| P to water | kg | 5.2 | 9.0 | - | - | - | - |
| Methane, biogenic | kg | - | - | 22.3 | 249.0 | 18.5 | 0.6 |
| N_2_O | kg | - | - | 0.1 | 0.9 | 0.4 | 0.0 |
| NH_3_ | kg | - | - | 3.8 | 39.3 | 13.2 | 13.1 |

^1)^ Data based on salmon aquaculture in Norway (Pelletier et al., 2009)

^2)^ Data based on shrimp aquaculture in China (Cao et al., 2012)

^3)^ Already published in Notarnicola et al. (2017)

## **Processing**

Data sources of processing phases of the new added products are presented in Table S4. Data in the study have been modified to the average EU situation, if the scope of the study was outside Europe, but processing phase was assumed to occur in Europe, e.g. tofu processing in the Mejia et al. (2017) was in USA, but energy consumption was changed to be average European consumption in this study.

**Table S4.** Data sources used in the modelling of processing phase of the new products added to BoP food.

| **Representative products** | **Activities** | **Data source** |
| --- | --- | --- |
| Wild cod | - Fishing of cod  - Processing of cod fillets | Svanes et al. (2011) |
| Farmed salmon | - Slaughtering  - Processing of salmon fillets | Elingsen et al. (2009) |
| Shrimps | - Processing of shrimps | Cao et al. (2011) |
| Rice | - Processing of rice | Blengini and Busto (2009) |
| Tofu | - Production of tofu | Mejia et al. (2017) |
| Bananas | - Post-harvest handling | Iriarte et al. (2014), Dole (2011) |
|  | - Ripening of bananas | Svanes and Aronsson (2013) |
| Almonds | - Processing of almonds | Kendall et al. (2015) |
| Tea | - Processing of tea | Jefferies et al. (2012) |
| Biscuits | - Baking of biscuits | Noya et al. (2018) |
| Chocolate | - Processing of cocoa | Ntiamoah and Afrane (2008) |
|  | - Production of chocolate | Recanati et al. (2018) |

## **Packaging**

Packaging types and amounts are presented in Tables S5 and S6. They were mainly obtained from the LCA studies used as data sources for modelling agricultural and/or processing stages, with exception of beans and almonds, for which the information was not available. Thus, the package type and amount for beans and almonds was estimated according to other food products. It was assumed that beans and almonds are packed into plastic bags with the same weight as plastic package used in the tofu packaging (according to Mejia et al. 2017). In addition, tomato packaging amount was based on Cellura et al. (2012) and egg packaging was based on Sonesson et al. (2008).

**Table S5.** Amounts of packaging per typology of products selected in this study (grams per 1 kg packaged product).

| **Product** | **Cardboard** | **Corrugated board box** | **Kraft paper** | **Cellulose fiber** | **Aluminium** | **LDPE** | **PS** |
| --- | --- | --- | --- | --- | --- | --- | --- |
| Cod | 100 | - | - | - | - | - | - |
| Salmon | - | - | - | - | - | - | 25 |
| Shrimps | 135 | - | - | - | - | 10.5 | - |
| Eggs | 69 | - | - | - | - | - | - |
| Rice | 50 | - | - | - | - | 10 | - |
| Tomatoes | - | 89.2 | - | - | - |  | - |
| Beans | - | - | - | - | - | 39 | - |
| Tofu | 93 | - | - | - | - | 39 | - |
| Bananas | - | 112.3 | - | - | - | 3.3 | - |
| Almonds | - | - | - | - | - | 39 | - |
| Tea | 260 | 280 | 440 | 10 | - | 42 | - |
| Biscuits | 170 | - | - | - | - | 5 | - |
| Chocolate | 118 | - | - | - | 18 | - | - |

**Table S6.** Amounts of packaging per typology of original products (grams per 1 kg packaged product) (already published in Notarnicola et al. (2017) and Castellani et al. (2018).

| **Product** | **Unit** | **Glass** | **Paper** | **Cardboard** | **Corrugated board box** | **Aluminium** | **LDPE** | **HDPE** | **PET** | **PP** | **PS** |
| --- | --- | --- | --- | --- | --- | --- | --- | --- | --- | --- | --- |
| Mineral water* | g | - | - | - | - | - | - | - | 23 | - | - |
| Beer | g | 522 | - | 32 | - | 3 | - | - | - | - | - |
| Wine** | g | 700 | - | - | 58 | - | - | - | - | - | - |
| Coffee - soluble | g | 2 600 | 4 | - | 54 | 14 | - | - | - | - | - |
| Coffee - ground | g | - | - | - | 14 | 16 | - | - | - | - | - |
| Apples*** | g | - | - | - | - | - | - | - | - | - | 3 |
| Oranges | g | - | - | - | 84 | - | - | - | - | - | - |
| Potatoes - fresh | g | - | - | - | - | - | - | 4 | - | - | - |
| Potatoes- frozen | g | - | - | - | - | 4 | - | - | 8 | - | - |
| Potatoes - chips | g | - | - | - | - | 20 | - | - | 20 | - | - |
| Bread | g | - | - | - | - | - | - | - | - | 4 | - |
| Pasta | g | - | - | 6 | 40 | - | 11 | - | - | - | - |
| Olive oil | g | 786 | 7 | - | 47 | 6 | 8 | - | - | - | - |
| Sunflower oil | g | - | - | - | 24 | - | - | - | 43 | - | - |
| Sugar | g | - | 15 | - | - | - | - | - | - | - | - |
| Milk* | g | - | - | - | - | - | - | - | 28 | - | - |
| Cheese | g | - | - | - | 115 | - | - | - | - | - | - |
| Butter | g | - | - | - | - | 15 | - | - | - | - | - |
| Beef | g | - | - | - | - | - | 4 | - | - | - | 33 |
| Pork | g | - | - | - | - | - | 4 | - | - | - | 33 |
| Poultry | g | - | - | - | - | - | 4 | - | - | - | 33 |
| Pre-prepared meal | g | - | - | - | 42 | - | 28 | - | 69 | 8 | - |
| * referred to as 1-L product | | | ** referred to as 0.75-L product | | | *** only 20% of product is packed | | | | | |

## **Logistics**

The transport of imported products was assumed to occur from the capital of the exporting country to the city of Frankfurt, which was considered a central destination for the arrival of imports in Europe. The transport was considered to be composed by: a transport by lorry between the capital of the exporting country and the country's main port; a transport by ship from the port of the exporting country to the main European ports and, finally, a transport by lorry between the port of destination and the city of Frankfurt. Rotterdam and Marseilles were considered as the European ports of arrival of the goods. The distances were calculated by using www.sea-distances.org and Google maps (Table S7 and S8). Sea and road transport was allocated to a percentage of the final product in the LCI model, corresponding to the share of imported goods out of the total apparent consumption of that kind of product.

**Table S7.** Summary of the share of imported food products, and sea and road transport distances of the products selected in this study.

| **Product** | **Import (%)** | **Sea transport (t*km) per kg of product imported** | **Road transport (t*km) per kg of product imported** |
| --- | --- | --- | --- |
| Fish (cod and salmon) | 66.9% | 4.62 | 0.57 |
| Shrimps | 64.7% | 10.42 | 1.12 |
| Eggs | 0.1% | 2.35 | 1.26 |
| Rice | 28.4% | 9.44 | 1.44 |
| Tomatoes | 7.7% | 1.87 | 0.53 |
| Beans | 16.5% | 3.76 | 0.84 |
| Soy beans for tofu | 92.8% | 7.13 | 2.16 |
| Bananas | 87.4% | 9.37 | 0.79 |
| Almonds | 35.8% | 6.27 | 0.68 |
| Tea | 100% | 10.48 | 1.46 |
| Wheat for biscuits | 4.2% | 2.19 | 0.29 |
| Palm oil for biscuits | 100% | 12.83 | 1.04 |
| Sugar for biscuits and chocolate | 4.5% | 0.43 | 0.10 |
| Cocoa beans for chocolate | 100% | 7.26 | 0.99 |

**Table S8.** Summary of the share of imported food products, and sea and road transport distances of the original products (already published in Castellani et al. (2018).

| **Product** | **Import (%)** | **Sea transport (t*km) per kg of product imported** | **Road transport (t*km) per kg of product imported** |
| --- | --- | --- | --- |
| Pig meat | 0.11% | 7.28 | 0.45 |
| Beef meat | 2.94% | 9.87 | 0.95 |
| Poultry meat | 1.34% | 7.34 | 2.07 |
| Milk & Cream | 0.02% | 0.35 | 0.59 |
| Cheese | 0.97% | 6.08 | 0.19 |
| Butter | 1.96% | 18.25 | 0.61 |
| Bread (wheat) | 4.2% | 2.19 | 0.29 |
| Pasta | 0.72% | 5.85 | 1.12 |
| Sugar | 4.53% | 0.43 | 0.10 |
| Sunflower oil | 4.04% | 1.66 | 0.81 |
| Olive oil | 2.77% | 0.93 | 0.87 |
| Potatoes | 0.75% | 2.55 | 1.04 |
| Oranges | 11.83% | 8.76 | 0.92 |
| Apples | 7.11% | 12.4 | 0.88 |
| Green coffee | 100% | 7.78 | 1.57 |
| Roasted coffee | 1.76% | 0.40 | 0.49 |
| Beer | 0.71% | 7.31 | 1.02 |
| Wine | 11.12% | 13.09 | 0.80 |
| Mineral water | 0.18% | 0.19 | 1.29 |
| Meat based dishes | - | - | - |

## **Use phase**

The following specific energy consumption were considered for preparation of food at home (Foster et al., 2006; Jefferies et al. 2012):

- Boiling: 2 MJ of natural gas/kg product (coffee, potatoes, eggs)
- Boiling of pre-soaked beans: 5.5 MJ of natural gas/kg product
- Boiling of rice: 1.7 MJ of natural gas/kg rice
- Frying: 7.5 MJ of natural gas/kg product (potatoes, fish, tofu, eggs)
- Roasting: 8.5 MJ of natural gas/kg product (meat products)
- Baking: 0.75 kWh electricity/kg product (potatoes)
- Cooking of pasta: 0.5 kWh electricity/kg and 2.3 MJ natural gas/kg
- Cooking of pre-prepared meal: 0.3 kWh electricity/meal
- Boiling of tea: 49.5 MJ of natural gas/kg tea.

Refrigerated storage at home was included in the life cycle of beer (14 days), butter (4 days), meat, milk, salmon and tofu (2 days), and frozen storage for potatoes, cod and shrimps (10 days). The electricity consumption of the domestic refrigerator was assumed to be 0.0023 kWh/L per day and the electricity consumption of the freezer is assumed to be 0.0042 kWh/L per day (Nielsen et al. 2003).

## **End-of-life**

Food waste amounts and data sources are presented in Table S9. The estimation of food waste generated in the different production stages was mainly based on FAO (2011). However, product specific data for processing stage presented in LCA literature was used when available, i.e. Kendall et al. (2015) for almonds, Pelletier et al. (2013) for eggs and Noya et al. (2018) for biscuits. For eggs, chocolate and biscuits, the food waste amount from household was not available in FAO (2011), hence WRAP (2014) data was used. In case of tofu, the food waste amount was not available at any stages, thus the waste generation was assumed to be equal to the one for oilseed and pulses reported by FAO (2011).

**Table S9.** Food waste amounts, expressed as percentage, in different life cycle stages for products selected in this study. Processing includes also waste from post-harvest selection.

| **Product** | **Processing** | **Logistics and retail** | **Household** | **Sources** |
| --- | --- | --- | --- | --- |
| **Fish** | 6% | 9% | 11% | FAO 2011 (fish) |
| **Shrimp** | 6% | 9% | 11%  (+15%) | FAO 2011 (fish),  In addition shells 15% |
| **Eggs** | 1.1% | 4% | 6.5%  (+ 16.6%) | Processing Pelletier et al. 2013;  Retail FAO 2011 (meat values used),  Household WRAP 2014, in addition shells 16.6% |
| **Tofu** | 5% | 1% | 4% | FAO 2011 (oilseed & pulses values used) |
| **Beans** | 5% | 1% | 4% | FAO 2011 (oilseed & pulses) |
| **Rice** | 10.5% | 2% | 25% | FAO 2011 (cereals) |
| **Bananas** | 5% | 10% | 19%  (+ 34%) | FAO 2011 (fruits & vegetables),  in addition peels 34% |
| **Tomatoes** | 5% | 10% | 19% | FAO 2011 (fruits & vegetables) |
| **Almonds** | 7.5% | 2% | 4% | Processing Kendall et al. 2015  Others FAO 2011 (cereal value used) |
| **Chocolate** | 5% | 1% | 4.4% | FAO 2011 (oilseed & pulses values used)  Household WRAP 2014 |
| **Biscuits** | 2.3% | 2% | 4.3% | Processing Noya et al. 2018  Retail FAO 2011 (cereals)  Household WRAP 2014 |
| **Tea** | 10.5% | 2% | 33.3%%  (+100%) | Processing and retail FAO 2011 (cereal values),  100% of tea leaves to waste after use,  1/3 cup of ready tea wasted |

# **Additional information on the results**

## **Midpoint results of EU food consumption**

- - 1. **Flow contribution to the overall environmental impact**

Table S10 presents the contribution by elementary flows of each impact category and the main processes behind the main elementary flow (market with *). The system under study is the same as in the paper, namely the one including recycling and reuse processes in the End of Life phase.

**Table S10**. Elementary flows and processes contributing most to the main elementary flow (marked with *) of BoP Food, expressed as percentage, for each impact category, according to the EF 2017 method.

| **Climate change** | | **Human tox, non-cancer effects** | | **Particulate matter** | |
| --- | --- | --- | --- | --- | --- |
| *Elementary flow* | *Contr.* | *Elementary flow* | *Contr.* | *Elementary flow* | *Contr.* |
| Methane, biogenic* | 29.6% | Mercury to soil* | 21.1% | Ammonia* | 81.9% |
| Carbon dioxide, fossil | 23.7% | Zinc to soil | 17.5% | Partic., <2.5 µm | 9.7% |
| CO_2_, land transformation | 15.1% | Arsenic to water | 16.5% | Partic., <10 µm | 3.4% |
| Dinitrogen monoxide | 11.8% | Lead to soil | 15.1% | Sulfur dioxide | 3.2% |
| *Process* | *Contr.* | *Process* | *Contr.* | *Process* | *Contr.* |
| Beef cattle for slaughter | 46.2% | Grass silage, beef farm | 26.9% | Beef cattle for slaughter | 28.2% |
| Raw milk, at farm | 29.1% | Grass silage, dairy farm | 21.9% | Pigs to slaughter | 23.3% |
| Pigs to slaughter | 11.6% | Maize silage, dairy farm | 14.2% | Raw milk, at farm | 21.4% |
| **Ozone depletion** | | **Human tox, cancer effects** | | **Ionizing radiation** | |
| *Elementary flow* | *Contr.* | *Elementary flow* | *Contr.* | *Elementary flow* | *Contr.* |
| CFC-113* | 50.0% | Chromium VI to water* | 48.8% | Radon-222 to air* | 55.3% |
| HFC-143a | 45.5% | Chromium to water | 31.5% | Carbon-14 to air | 40.5% |
| Halon 1301 | 1.5% | Chromium to soil | 11.7% | Cesium-137 to water | 2.6% |
| *Process* | *Contr.* | *Process* | *Contr.* | *Process* | *Contr.* |
| Refrigerant R404a | 99.8% | Steel, low-alloyed, at plant | 23.7% | Electricity production | 68.9% |
|  |  | Cast-iron {GLO}, market for | 9.8% | Aluminium, wrought alloy {GLO}, market for | 1.0% |
| **Photochemical ozone formation** | | **Acidification** | | **Terrestrial eutrophication** | |
| *Elementary flow* | *Contr.* | *Elementary flow* | *Contr.* | *Elementary flow* | *Contr.* |
| Nitrogen oxides* | 69.5% | Ammonia* | 86.4% | Ammonia* | 90.7% |
| Nitrogen dioxide | 8.5% | Sulfur dioxide | 6.6% | Nitrogen oxides | 8.3% |
| NMVOC, unsp. origin | 7.7% | Nitrogen oxides | 6.1% | Nitrogen dioxide | 1.0% |
| *Process* | *Contr.* | *Process* | *Contr.* | *Process* | *Contr.* |
| Diesel use in machinery | 24.4% | Beef cattle for slaughter | 28.2% | Beef cattle for slaughter | 28.2% |
| Transport, freight, lorry | 17.9% | Pigs to slaughter | 23.3% | Pigs to slaughter | 23.3% |
| Cod fishing | 12.5% | Raw milk, at farm | 21.4% | Raw milk, at farm | 21.4% |

**TableS10 (continue)**

| **Freshwater eutrophication** | | **Marine eutrophication** | | **Water use** | |
| --- | --- | --- | --- | --- | --- |
| *Elementary flow* | *Contr.* | *Elementary flow* | *Contr.* | *Elementary flow* | *Contr.* |
| Phosphorus, total to water* | 31.4% | Nitrate to water* | 63.9% | Water balance in CN | 22.4% |
| Phosphate to water | 24.6% | Nitrogen, total to water | 21.7% | Water balance in ES | 15.7% |
| Fertiliser, applied (P component) to soil | 24.3% | Nitrogen oxides to air | 7.0% | Water balance in IT | 10.5% |
| Manure, applied (P component) to soil | 13.9% | Ammonia to air | 5.8% | Water balance in unspecified country | 9.7% |
| *Process* | *Contr.* | *Process* | *Contr.* | *Process* | *Contr.* |
| Wastewater treatment | 72.5% | Grass, at beef farm | 12.0% | Harvested wine grapes | 33.2% |
| Salmon aquaculture | 15.4% | Grass, at dairy farm | 10.5% | Rice cultivation | 12.0% |
| Shrimp aquaculture | 6.9% | Wheat grain | 8.1% | Almond cultivation | 4.2% |
| **Land transformation** | | **Land occupation** | | **Freshwater ecotoxicity** | |
| *Elementary flow* | *Contr.* | *Elementary flow* | *Contr.* | *Elementary flow* | *Contr.* |
| From forest to arable* | 67.5% | Occupation, arable* | 95.5% | Chlorpyrifos to soil* | 22.5% |
| From grassland to arable | 5.1% | Occupation, permanent crop | 2.2% | Copper to soil | 17.5% |
| *Process* | *Contr.* | *Process* | *Contr.* | Zinc to soil | 12.8% |
| Soybean, at farm | n.a. | Soybean, at farm | 12.0% | Folpet to soil | 10.6% |
|  |  | Grass, at beef farm | 7.6% | Zinc to water | 3.8% |
|  |  | Grass, at dairy farm | 5.9% | Chlorothalonil to soil | 3.0% |
| **Resource use, minerals and metals** | | **Resource use, fossil** | | Antimony to air | 2.4% |
| *Elementary flow* | *Contr.* | *Elementary flow* | *Contr.* | Chromium to water | 2.4% |
| Cadmium* | 23.2% | Oil, crude* | 34.7% | Pretilchlor to soil | 2.2% |
| Lead | 16.9% | Natural gas | 33.8% | Cyfluthrin to soil | 2.0% |
| Gold | 13.2% | Coal, hard | 13.8% |  |  |
| *Process* | *Contr.* | *Process* | *Contr.* | *Process* | *Contr.* |
| Zinc-lead mining | 38.0% | Transportation, lorry | 35.8% | Soybean meal | 19.9% |
| Passenger car | 18.1% | Passenger car | 10.5% | Sunflower seed, at farm | 16.2% |
| Transport, freight, lorry | 12.1% | Cod fishing | 10.1% | Cocoa cultivation | 14.2% |

n.a. – not available

- - 1. **Results per kg food product**

Table S11 presents the impact assessment results of representative food products per kg food, including recycling and reuse processes in the End of Life phase.

**Table S11.** Midpoint results per kg food.

### **Comparison of results: system with vs system without reuse and recycling**

Table S12 presents the results of the impact assessment of the BoP Food (characterized by means of the EF 2017 method) without taking into account recycling and reuse compared to the results taking those into account. In general, when recycling and reuse are taken into account, the environmental impacts are lower in almost all impact categories. Only freshwater ecotoxocity shows higher impact (1%) when recycling and reuse are included to the assessment.

**Table S12.** Comparison of characterized midpoint results of food consumption by an average EU-28 citizen without and with recycling and reuse in 2015.

| **Impact category** | **Unit** | **Food consumption without recycling** | **Food consumption with recycling** | **Difference, %** |
| --- | --- | --- | --- | --- |
| Climate change | kg CO_2_ eq | 2.78E+3 | 2.72E+3 | -2% |
| Ozone depletion | kg CFC-11 eq | 3.50E-3 | 3.51E-3 | 0% |
| Human toxicity, non-cancer | CTUh | 1.66E-4 | 1.61E-4 | -3% |
| Human toxicity, cancer | CTUh | 3.16E-5 | 3.03E-5 | -4% |
| Particulate matter | Disease incidence | 2.95E-4 | 2.83E-4 | -4% |
| Ionising radiation | kBq U235 eq | 5.46E+1 | 5.36E+1 | -2% |
| Photochemical ozone formation | kg NMVOC eq | 4.95E+0 | 4.60E+0 | -8% |
| Acidification | molc H^+^ eq | 3.94E+1 | 3.87E+1 | -2% |
| Terrestrial eutrophication | molc N eq | 1.66E+2 | 1.65E+2 | -1% |
| Freshwater eutrophication | kg P eq | 6.92E-1 | 6.90E-1 | 0% |
| Marine eutrophication | kg N eq | 1.76E+1 | 1.75E+1 | -1% |
| Ecotoxicity freshwater | CTUe | 7.19E+3 | 7.25E+3 | +1% |
| Land use | Pt | 2.69E+5 | 2.62E+5 | -3% |
| Water scarcity | m^3^ water eq | 5.14E+3 | 5.09E+3 | -1% |
| Resource use (fossils) | MJ | 1.69E+4 | 1.56E+4 | -8% |
| Resource use (mineral and metals) | kg Sb eq | 2.44E-3 | 2.45E-3 | 0% |

## **Endpoint results of EU food consumption: impacts on biodiversity**

### **Flow contribution to biodiversity loss**

Tables S13 and S14 report the three most relevant elementary flows behind the BoP Food for each impact category, according to ReCiPe 2008 and ReCiPe 2016 respectively. Within each impact category, for the flow that contributes the most, the main process from which it originates is specified (marked with *). The system under study is the same as in the manuscript, namely it includes recycling and reuse processes in the End of Life phase.

**Table S13.** Elementary flow contribution to biodiversity loss, expressed as percentage, for each impact category, according to ReCiPe 2008.

| **Climate change Ecosystems** | | **Terrestrial acidification** | | **Freshwater eutrophication** | |
| --- | --- | --- | --- | --- | --- |
| *Elementary flow* | *Contr.* | *Elementary flow* | *Contr.* | *Elementary flow* | *Contr.* |
| Methane, biogenic* | 48.69% | Ammonia* | 94.17% | Fertiliser, applied  (P component) to soil* | 45.65% |
| Dinitrogen monoxide | 19.72% | Nitrogen oxides | 2.99% | Phosphorus, total to water | 26.77% |
| Carbon dioxide, fossil | 12.11% | Sulfur dioxide | 2.25% | Manure, applied  (P component) to soil | 21.13% |
| * Beef cattle for slaughter | | * Beef cattle for slaughter | | * Soybean cultivation | |
| **Terrestrial ecotoxicity** | | **Freshwater ecotoxicity** | | **Marine ecotoxicity** | |
| *Elementary flow* | *Contr.* | *Elementary flow* | *Contr.* | *Elementary flow* | *Contr.* |
| Cypermethrin to soil* | 88.02% | Chlorine to water* | 75.39% | Chlorine to water* | 90.04% |
| Isoproturon to soil | 3.60% | Cypermethrin to soil | 17.22% | Copper to air | 5.57% |
| Azoxystrobin to soil | 2.56% | Azoxystrobin to soil | 1.60% | Cypermethrin to soil | 1.09% |
| * Soybean cultivation | | * Wastewater treatment | | * Wastewater treatment | |
| **Agricultural land occupation** | | **Urban land occupation** | | **Natural land transformation** | |
| *Elementary flow* | *Contr.* | *Elementary flow* | *Contr.* | *Elementary flow* | *Contr.* |
| Occupation, arable* | 99.88% | Occupation, traffic area, road network* | 62.49% | Transformation, from forest* | 99.64% |
| Occupation, forest, intensive | 0.10% | Occupation, industrial area | 13.78% | Transformation, from forest, intensive | 0.73% |
| Occupation, permanent crop | 0.01% | Occupation, traffic area, rail/road embankment | 10.25% | Transformation, from forest, extensive | 0.53% |
| * Grass, grazed in pasture | | * Transport, freight, lorry | | * Soybean cultivation | |

**Table S14.** Elementary flow contribution to biodiversity loss, expressed as percentage, for each impact category, according to ReCiPe 2016

| **Global warming,**  **Terrestrial ecosystems** | | **Global warming,**  **Freshwater ecosystems** | | **Ozone formation,**  **Terrestrial ecosystems** | |
| --- | --- | --- | --- | --- | --- |
| *Elementary flow* | *Contr.* | *Elementary flow* | *Contr.* | *Elementary flow* | *Contr.* |
| Methane, biogenic* | 58.87% | Methane, biogenic* | 58.84% | Nitrogen oxides* | 97.85% |
| Dinitrogen monoxide | 15.59% | Dinitrogen monoxide | 15.59% | NMVOC | 1.95% |
| Carbon dioxide, fossil | 9.54% | Carbon dioxide, fossil | 9.56% | Pentane | 0.04% |
| * Beef cattle for slaughter | | * Beef cattle for slaughter | | * Fishing vessel, diesel combusted | |
| **Terrestrial acidification** | | **Freshwater eutrophication** | | **Terrestrial ecotoxicity** | |
| *Elementary flow* | *Contr.* | *Elementary flow* | *Contr.* | *Elementary flow* | *Contr.* |
| Ammonia* | 94.22% | Fertiliser, applied  (P component) to soil | 54.59% | Copper to air* | 80.99% |
| Sulfur dioxide | 2.80% | Manure, applied  (P component) to soil | 26.75% | Zinc to air | 8.16% |
| Nitrogen oxides | 2.40% | Phosphorus, total to water | 16.94% | Copper to soil | 2.24% |
| * Beef cattle for slaughter | | * Soybean cultivation | | * Heat production (light fuel oil) | |

**Table S14 (continue)**

| **Freshwater ecotoxicity** | | **Marine ecotoxicity** | | **Land use** | |
| --- | --- | --- | --- | --- | --- |
| *Elementary flow* | *Contr.* | *Elementary flow* | *Contr.* | *Elementary flow* | *Contr.* |
| Chlorpyrifos to soil* | 44.51% | Zinc to water* | 28.59% | Occupation, arable* | 99.89% |
| Zinc to water | 15.52% | Chlorpyrifos to soil | 17.11% | Occupation, forest, intensive | 0.05% |
| Cypermethrin to soil | 4.74% | Chlorothalonil to soil | 11.26% | Occupation, traffic area, road network | 0.02% |
| * Soybean production | | * Soybean production | | * Grass grazed in pasture | |
| **Water consumption,**  **Terrestrial ecosystem** | | **Water consumption,**  **Aquatic ecosystems** | |  |  |
| *Elementary flow* | *Contr. ^§^* | *Elementary flow* | *Contr. ^§^* |  |  |
| Water, turbine use, unspecified natural origin | 93.57% | Water, turbine use, unspecified natural origin | 93.57% |  |  |
| Water, turbine use, unspecified natural origin, SE | 44.06% | Water, turbine use, unspecified natural origin, SE | 44.06% |  |  |
| Water, turbine use, unspecified natural origin, FR | 31.13% | Water, turbine use, unspecified natural origin, FR | 31.13% |  |  |
| * Steel, low-alloyed, at plant/RER S | | * Steel, low-alloyed, at plant/RER S | |  |  |
| § contribution is >100% since there is a benefit from reuse of water (negative values) that balances the % | | § contribution is >100% since there is a benefit from reuse of water (negative values) that balances the % | |  |  |

### **Land use analysis**

Table S15 and S16 report the relative importance of each products to the damage to ecosystem quality, based on the two implemented versions of the method by Chaudhary et al. (2015). Specifically, the first version characterizes only the highest land use level according to the original flow mapping of Chaudhary et al. (2015), while in the second version all the sub-types of land use (i.e. second and third level according to Koellner et al. 2013) were mapped by using the CF of the highest level, in order to broaden the coverage of the inventory.

The system under study is the same as in the manuscript, namely it includes recycling and reuse processes in the End of Life phase.

**Table S15.** Relative importance of each product to the damage on ecosystem quality due to land use in the BoP Food, based on the original impact assessment model and factors of Chaudhary et al. (2015)

**Table S16.** Relative importance of each product to the damage on ecosystem quality due to land use in the BoP Food, based on the impact assessment model of Chaudhary et al. (2015) remapped in order to broaden the coverage of the inventory

### **Biodiversity impact assessment of the BoP Food, without accounting for reuse and recycling**

In line with the presentation of the results in section 3.2 of the manuscript, Tables S17 and S18 present the endpoint results for the Area of Protection “Ecosystem quality” (representative of the damage to the natural systems and biodiversity) of the BoP Food system, in which benefits from recycling and reuse within the End of Life stage are not taken into account. Both ReCiPe 2008 and ReCiPe 2016 were applied.

The results, both for the food consumption at EU-28 level and per capita, indicate that three impact categories mainly contribute to more than 80% of the total impact of the BoP Food in terms of loss of species. In ReCiPe 2008 these categories are related to land use and land use change (i.e. agricultural land occupation and natural land transformation) and climate change. In ReCiPe 2016 as well, land use and global warming in terrestrial ecosystems play a crucial role in determining biodiversity decline; these categories are followed by terrestrial acidification.

**Table S17.** Results of the endpoint characterization of the BoP Food, both for consumption at EU-28 level and per capita, by means of ReCiPe 2008. The endpoint results are reported only for the Area of Protection “Ecosystem quality”, which reflects the impacts on biodiversity.

| **Impact category** | **Abbreviations** | **Unit** | **Result for EU-28 consumption** | **Result for an average EU citizen** | **Impact of EU consumption on ecosystem quality (% of species loss)** |
| --- | --- | --- | --- | --- | --- |
| Climate change Ecosystems | CC | species.yr | 1.00E+04 | 1.99E-05 | 25.21% |
| Terrestrial acidification | AC | species.yr | 9.34E+01 | 1.85E-07 | 0.23% |
| Freshwater eutrophication | FEU | species.yr | 1.61E+01 | 3.18E-08 | 0.04% |
| Terrestrial ecotoxicity | ECOTOX-T | species.yr | 1.91E+03 | 3.78E-06 | 4.79% |
| Freshwater ecotoxicity | ECOTOX-F | species.yr | 1.82E+01 | 3.61E-08 | 0.05% |
| Marine ecotoxicity | ECOTOX-M | species.yr | 6.69E-01 | 1.32E-09 | 0.00% |
| Agricultural land occupation | ALO | species.yr | 2.01E+04 | 3.98E-05 | 50.51% |
| Urban land occupation | ULO | species.yr | 1.17E+02 | 2.31E-07 | 0.29% |
| Natural land transformation | NLT | species.yr | 7.52E+03 | 1.49E-05 | 18.87% |
| **Total Land Use impact** | **-** | **species.yr** | **2.78E+04** | **5.50E-05** | **69.67%** |
| **Total Ecosystem Quality Impact** | **-** | **species.yr** | **3.98E+04** | **7.89E-05** | **100.00%** |

**Table S18.** Results of the endpoint characterization of the BoP Food, both for consumption at EU-28 level and per capita, by means of ReCiPe 2016. The endpoint results are reported only for the Area of Protection “Ecosystem quality”, which reflects the impacts on biodiversity.

| **Impact category** | **Abbreviations** | **Unit** | **Result for EU-28 consumption** | **Result for an average EU citizen** | **Impact of EU consumption on ecosystem quality (% of species loss)** |
| --- | --- | --- | --- | --- | --- |
| Global warming, Terrestrial ecosystems | CC-T | species.yr | 4.01E+03 | 7.93E-06 | 23.19% |
| Global warming, Freshwater ecosystems | CC-F | species.yr | 1.09E-01 | 2.17E-10 | 0.00% |
| Ozone formation, Terrestrial ecosystems | POF-T | species.yr | 2.37E+02 | 4.70E-07 | 1.37% |
| Terrestrial acidification | AC | species.yr | 2.75E+03 | 5.44E-06 | 15.91% |
| Freshwater eutrophication | FEU | species.yr | 3.16E+02 | 6.26E-07 | 1.83% |
| Terrestrial ecotoxicity | ECOTOX-T | species.yr | 2.31E+01 | 4.58E-08 | 0.13% |
| Freshwater ecotoxicity | ECOTOX-F | species.yr | 4.06E+00 | 8.04E-09 | 0.02% |
| Marine ecotoxicity | ECOTOX-M | species.yr | 4.13E-01 | 8.18E-10 | 0.00% |
| Land use | LU | species.yr | 8.99E+03 | 1.78E-05 | 52.04% |
| Water consumption, Terrestrial ecosystem | WU-T | species.yr | 9.47E+02 | 1.88E-06 | 5.48% |
| Water consumption, Aquatic ecosystems | WU-F | species.yr | 4.24E-02 | 8.39E-11 | 0.00% |
| **Total Ecosystem Quality impact** | **-** | **species.yr** |  |  | **100.00%** |

According to both the impact assessment methods, the three impact categories mentioned above, additionally to water consumption for ReCiPe 2016, contribute the most to biodiversity decline for each product, with land use related categories and climate change being generally the most impacting ones (Figure S1 and Figure S2). At product level, loss of species due to EU food consumption is driven by meat, specifically pork and beef.

**Figure S1.** Impact category contribution, expressed as percentage, to the impact to ecosystem quality by product, by applying ReCiPe 2008. Absolute results in terms of species lost per years are reported on top of each impact category.

**
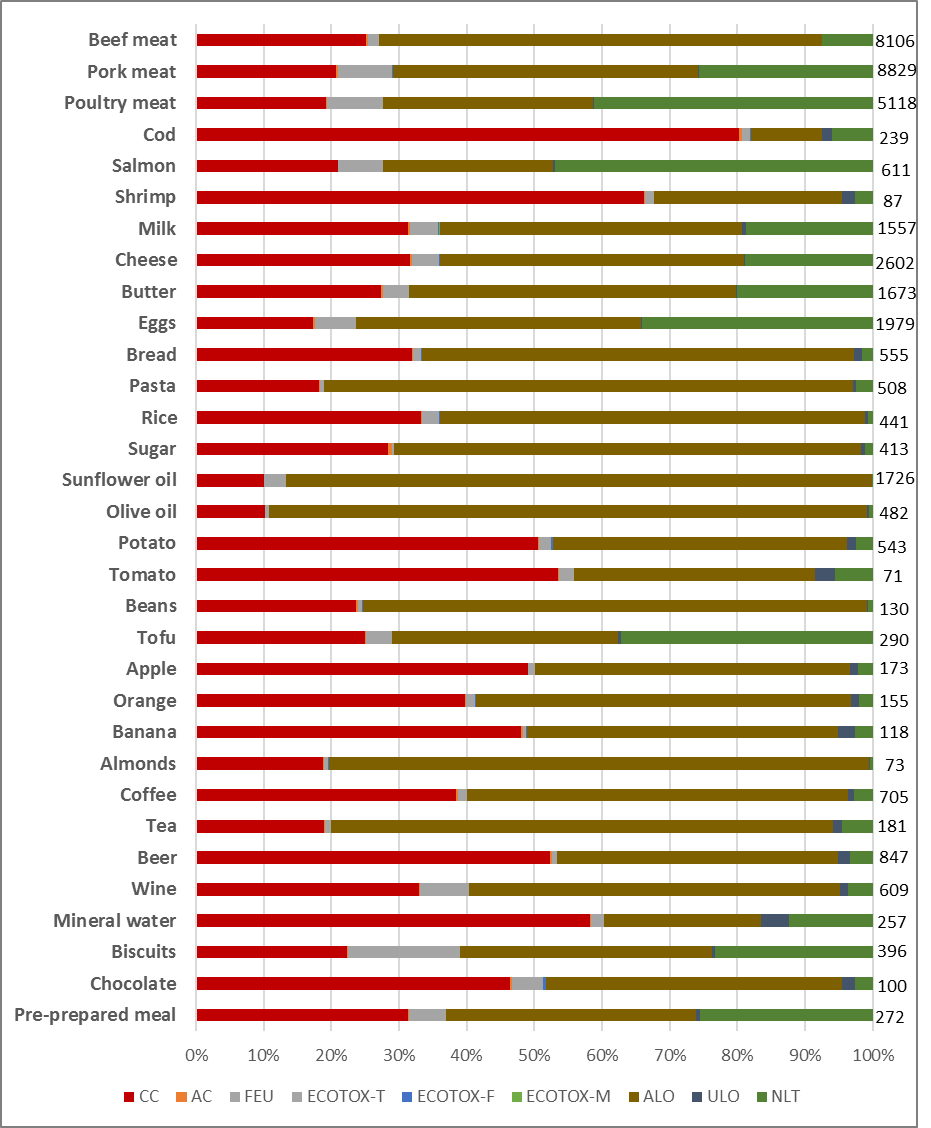
**

CC: Climate change Ecosystems; AC: Terrestrial acidification; FEU: Freshwater eutrophication; ECOTOX-T: Terrestrial ecotoxicity; ECOTOX-F: Freshwater ecotoxicity; ECOTOX-M: Marine ecotoxicity; ALO: Agricultural land occupation; ULO: Urban land occupation; NLT: Natural land transformation

**Figure S2.** Impact category contribution, expressed as percentage, to the impact to ecosystem quality by product, by applying ReCiPe 2016. Absolute results in terms of species lost per years are reported on top of each impact category.


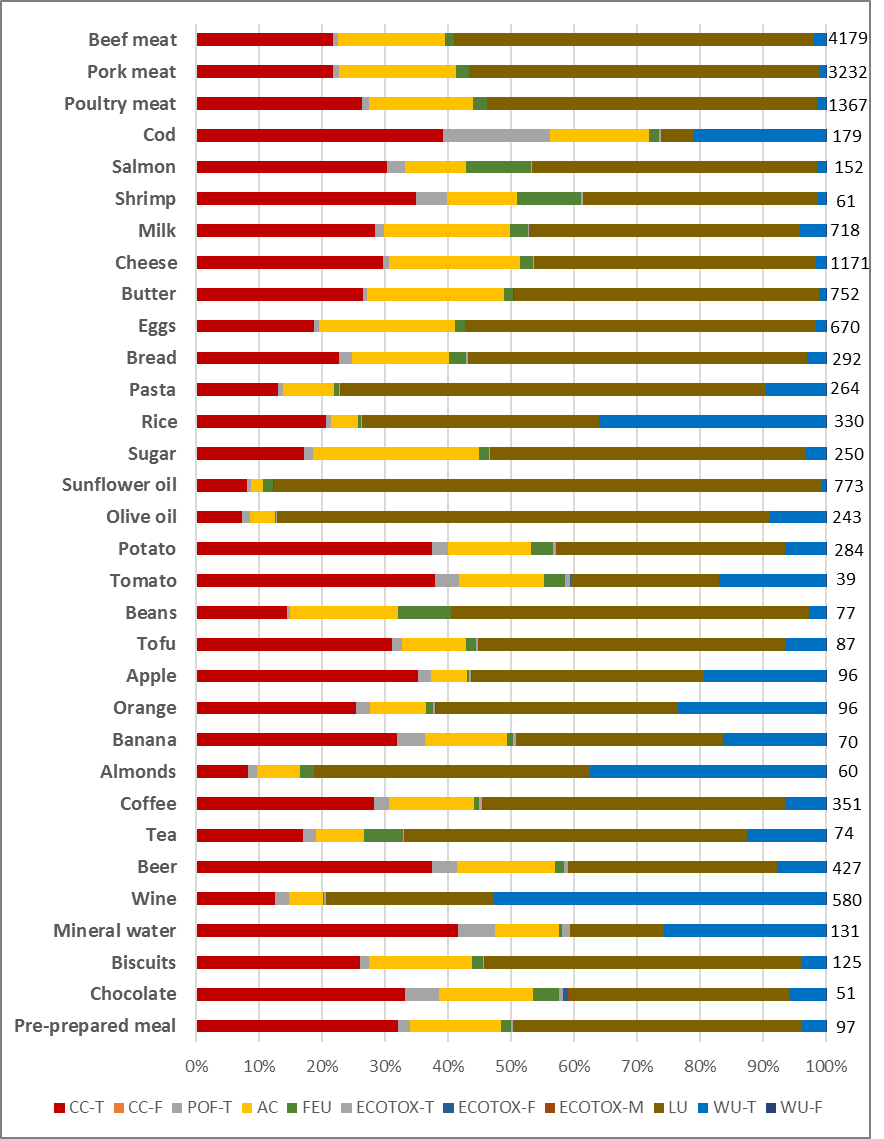


CC-T: Global warming, Terrestrial ecosystems; CC-F: Global warming, Freshwater ecosystems; POF-T: Ozone formation, Terrestrial ecosystems; AC: Terrestrial acidification; FEU: Freshwater eutrophication; ECOTOX-T: Terrestrial ecotoxicity; ECOTOX-F: Freshwater ecotoxicity; ECOTOX-M: Marine ecotoxicity; LU: Land use; WU-T: Water consumption, Terrestrial ecosystem; WU-F: Water consumption, Aquatic ecosystems

Generally the accounting of recycling and reuse in the end of life phase, especially for packaging, allows to avoid environmental impacts for a broad variety of food products, namely at least 20 products out of 32 for which a minor impact is evident (Table S19). Avoided impact per product spans from less than 1% as for instance in apples, potatoes and bread, up to 61% and 28% in tomatoes according to ReCiPe 2008 and ReCiPe 2016 respectively.

**Table S19**. Impact avoided, in terms of percentage, in the EU food consumption due to the inclusion of recycling and reuse in the End of Life phase of each representative product.

| **Products** | **ReCiPe 2008** | **ReCiPe 2016** |
| --- | --- | --- |
| Beef meat | 0% | 0% |
| Pork meat | 0% | 0% |
| Poultry meat | 0% | 0% |
| Cod | -14% | -5% |
| Salmon | 0% | 0% |
| Shrimps | -8% | -3% |
| Milk | -1% | -1% |
| Cheese | -2% | -1% |
| Butter | 0% | 0% |
| Eggs | -2% | -1% |
| Bread | 0% | 0% |
| Pasta | -9% | -5% |
| Rice | -3% | -1% |
| Sugar | -3% | -1% |
| Sunflower oil | 0% | 0% |
| Olive oil | -4% | -4% |
| Potato | 0% | 0% |
| Tomato | -61% | -28% |
| Beans | 0% | 0% |
| Tofu | -5% | -5% |
| Apple | 0% | 0% |
| Orange | -23% | -10% |
| Banana | -37% | -16% |
| Almonds | 0% | 0% |
| Coffee | -1% | -1% |
| Tea | -10% | -6% |
| Beer | -21% | -20% |
| Wine | -11% | -5% |
| Mineral water | -19% | -18% |
| Biscuits | -9% | -8% |
| Chocolate | -21% | -12% |
| Pre-prepared meal | 0% | 0% |

The method developed by Chaudhary et al. (2015) was also applied, by implementing in SimaPro v.8.5 two versions of the same method. Specifically, the original method covering six land use types (i.e. arable, permanent crops, pasture and meadow, urban, forest extensive and intensive) was firstly adopted. Then, an integrated version was applied, in which all the sub-types of land use flows were mapped by assigning the CF of the highest level as proposed in the original method, in order to broaden the coverage of the inventory.

By applying the original version of the method as proposed by Chaudhary et al. (2015), all the sets of CFs agree on that pork meat, followed by beef and poultry meat, represents the food product that mostly affects biodiversity (Table S20), being in line with ReCiPe 2008. Furthermore, shrimps appear to be the least impacting food product, with an apparent benefit (negative value of around -3%). However, since in this system the avoided impacts due to recycling are not accounted for, this negative result is associated to a limitation of the method itself, as explained in the manuscript. Specifically this result is due to a poor flow coverage, thus bringing to a misleading result. The situation changes by adopting the integrated version in which all the sub-types of land use were remapped (Table S21). In fact, shrimps turn to have an environmental impact on biodiversity, close to other products such as cod and tomatoes. It has to be considered that the remapping of flows at lower levels may lead to potential under- or overestimation of impacts. For example “occupation, forest, intensive, short-cycle” was mapped with the same CF as “occupation, forest, intensive”, which has a higher impact; similarly, “occupation, arable, irrigated, intensive” was mapped with the CF of “occupation, arable”, which has a lower environmental impact.

**Table S20**. Relative importance of each product to the damage on ecosystem quality due to land use in the BoP Food, based on the original impact assessment model and factors of Chaudhary et al. (2015). Results are referred to the system without reuse and recycling.

**Table S21.** Relative importance of each product to the damage on ecosystem quality due to land use in the BoP Food, based on the impact assessment model and factors of Chaudhary et al. (2015) remapped in order to broaden the coverage of the inventory. Results are referred to the system without reuse and recycling.

## **References**

Bartzas, G., Vamvuka, D. & Komnitsas, K. (2017). Comparative life cycle assessment of pistachio, almond and apple production. Information Processing in agriculture, 4: 188-198.

Blengini, G.A. & Busto, M. (2009). The life cycle of rice: LCA of alternative agri-food chain management systems in Vercelli (Italy). Journal of Environmental Management, 90: 1512-1522.

Blonk Consultants (2014). Agri-footprint 2.0. Description of Data. Retrieved from: http://www.agri-footprint.com/wp-content/uploads/2016/08/Agri-footprint-2.0-Part-2-Description-of-data.pdf (Accessed March 2018).

Cao, L., Diana, J.S., Keoleian, G.A. & Lai, Q. (2011). Life Cycle Assessment of Chinese Shrimp Farming Systems Targeted for Export and Domestic Sales. Environmental Science & Technology, 45: 6531-6538.

Castellani, V., Fusi, A. & Sala, S. (2017). Consumer Footprint. Basket of Products indicator on Food. JRC Technical Reports. Publications Office of the European Union, Luxemburg, 2017.

Cellura, M., Longo, S., Mistretta, M. (2012). Life Cycle Assessment (LCA) of protected crops: an Italian case study. Journal of Cleaner Production 28, 56-62.

Chapagain, A.K. & Hoekstra, A.Y. 2010. The green, blue and grey water footprint of rice from both a production and consumption perspective. Value of Water. Research Report Series No. 40. UNESCO-IHE. Institute for Water Education.

Chaudhary, A., Verones, F., de Baan, L., & Hellweg, S. (2015). Quantifying land use impacts on biodiversity: combining species–area models and vulnerability indicators. Environmental science & technology, 49(16): 9987-9995.

Dole (2011). Water Recycling Programs for Banana Packing. Water management. Dole corporate responsibility and sustainability. Available online: http://dolecrs.com/sustainability/water-management/water-recycling-programs-for-banana-packing/ (Accessed April 2018).

Ellingsen, H., Olaussen, J.O. & Utne, I.B. (2009). Environmental analysis of the Norwegian fishery and aquaculture industry – A preliminary study focusing on farmed salmon. Marine Policy, 33: 479-488.

FAO (2011). Global food losses and food waste – Extent, causes and prevention. Rome.

Foster, C., Green, K., Bleda, M., Dewick, P., Evans, B. Flynn, A. & Mylan J. (2006). Environmental Impacts of Food Production and Consumption: A report to the Department for Environment, Food and Rural Affairs. Manchester Business School. Defra, London.

Iriarte, A., Almeida, M.G. & Villalobos, P. (2014). Carbon footprint of premium quality export bananas: Case study in Ecuador, the world’s largest exporter. Science of the Total Environment, 472: 1082-1088.

Jefferies, D., Muñoz, I., Hodges, J., King, V.J., Aldaya, M., Ercin, A.E., Mila i Canals, L. & Hoekstra, A.Y. (2012). Water Footprint and Life Cycle Assessment as approaches to assess potential impacts of products on water consumption. Key learning points from pilot studies on tea and margarine. Journal of Cleaner Production, 33: 155-166.

Kendall, A., Marvinney, E., Brodt, S. & Zhu, W. (2015). Life Cycle-based Assessment of Energy Use and Greenhouse Gas Emissions in Almond Production, Part I. Analytical Framework and Baseline Results. Journal of Industrial Ecology, 19 (6): 1008-1018.

Mejia, A., Harwatt, H., Jaceldo-Siegl, K., Sranacharoenpong, K., Soret, S. & Sabate, J. (2017). Greenhouse Gas Emissions Generated bu Tofu Production: A Case Study. Journal of Hunger & Environmental Nutrition, 1-12.

Mekonnen, M.M. & Hoekstra, A.Y. (2010). The green, blue and grey water footprint of crops and derived crop products. Volume 2: Appendices.Value of Water. Research Report Series No. 47.UNESCO-IHE. Institute for Water Education.

Nielsen PH., Nielsen AM., Weidema BP., Dalgaard R. & Halberg N. (2003). LCA food database. www.lcafood.dk.

Notarnicola, B., Tassielli, G., Renzulli, P. A., Castellani, V., & Sala, S. (2017). Environmental impacts of food consumption in Europe. Journal of Cleaner Production, 140, 753-765.

Noya, L.I., Vasilaki, V., Stojceska, V., Gonzalez-Garcia, S., Kleynhans, C., Tassou, S., Moreira, M.T. & Katsou, E. (2018). An environmental evaluation of food supply chain using life cycle assessment: A case study on gluten free biscuit products. Journal of Cleaner Production, 170: 451-461.

Ntiamoah, A. & Afrane, G. (2008). Environmental impacts of cocoa production and processing in Ghana: life cycle assessment approach. Journal of Cleaner Production, 16: 1735-1740.

Pelletier, N., Ibarburu, M. & Xin, H. (2013). A carbon footprint analysis of egg production and processing supply chains in the Midwestern United States. Journal of Cleaner Production, 54: 108-114.

Pelletier, N., Tyedmers, P., Sonesson, U., Scholz, A., Ziegler, F., Flysjo, A., Kruse, S., Cancino, B. & Silverman, H. (2009). Not All Salmon Are Created Equal: Life Cycle Assessment (LCA) of Global Salmon Farming System. Environmental Science & Technology, 43: 8730-8736.

Recanati, F., Marveggio, D. & Dotelli, G. (2018). From beans to bar: A life cycle assessment towards sustainable chocolate supply chain. Science of the Total Environment, 613-614: 1013-1023.

Sonesson, U., Cederberg, C., Flysjö, A., Carlsson, B. (2008). Livscykelanalysis (LCA) av svenska ägg (ver.2). SIK-rapport Nr 783 2008.

Svanes, E. & Aronsson, K.S. (2013). Carbon footprint of a Cavendish banana supply chain. International Journal of Life Cycle Assessment, 18: 1450-1464.

Svanes, E., Vold, M. & Hanssen, O.J. (2011). Environmental Assessment of cod (Gadus morhua) from autoline fisheries. International Journal of Life Cycle Assessment, 16:611-624.

Torrellas, M., Anton, A., Lopez, J.C., Baeza, E.J., Parra, J.P., Muñoz, P. & Montero, J.I. (2012). LCA of a tomato crop in a multi-tunnel greenhouse in Almeria. International Journal of Life Cycle Assessment, 17: 863-875.

WRAP (2014). Household food and drink waste: A product focus. Final report. Waste & Resources Action Programme.
